# miR-126-5p by direct targeting of JNK-interacting protein-2 (JIP-2) plays a key role in *Theileria*-infected macrophage virulence

**DOI:** 10.1371/journal.ppat.1006942

**Published:** 2018-03-23

**Authors:** Malak Haidar, Zineb Rchiad, Hifzur Rahman Ansari, Fathia Ben-Rached, Shahin Tajeri, Perle Latre De Late, Gordon Langsley, Arnab Pain

**Affiliations:** 1 Pathogen Genomics Laboratory, Biological and Environmental Sciences and Engineering (BESE) Division, King Abdullah University of Science and Technology (KAUST), Thuwal, Kingdom of Saudi Arabia; 2 Inserm U1016, Cnrs UMR8104, Cochin Institute, Paris, France; 3 Laboratoire de Biologie Cellulaire Comparative des Apicomplexes, Faculté de Médecine, Université Paris Descartes - Sorbonne Paris Cité, Paris, France; 4 Global Station for Zoonosis Control, Global Institution for Collaborative Research and Education (GI-CoRE), Hokkaido University, N20 W10 Kita-ku, Sapporo, Japan; University of New Mexico, UNITED STATES

## Abstract

*Theileria annulata* is an apicomplexan parasite that infects and transforms bovine macrophages that disseminate throughout the animal causing a leukaemia-like disease called tropical theileriosis. Using deep RNAseq of *T*. *annulata*-infected B cells and macrophages we identify a set of microRNAs induced by infection, whose expression diminishes upon loss of the hyper-disseminating phenotype of virulent transformed macrophages. We describe how infection-induced upregulation of miR-126-5p ablates JIP-2 expression to release cytosolic JNK to translocate to the nucleus and trans-activate AP-1-driven transcription of *mmp9* to promote tumour dissemination. In non-disseminating attenuated macrophages miR-126-5p levels drop, JIP-2 levels increase, JNK1 is retained in the cytosol leading to decreased c-Jun phosphorylation and dampened AP-1-driven *mmp9* transcription. We show that variation in miR-126-5p levels depends on the tyrosine phosphorylation status of AGO2 that is regulated by Grb2-recruitment of PTP1B. In attenuated macrophages Grb2 levels drop resulting in less PTP1B recruitment, greater AGO2 phosphorylation, less miR-126-5p associated with AGO2 and a consequent rise in JIP-2 levels. Changes in miR-126-5p levels therefore, underpin both the virulent hyper-dissemination and the attenuated dissemination of *T*. *annulata*-infected macrophages.

## Author summary

*Theileria annulata*-infected bovine macrophages lose their hyper-disseminating virulent phenotype during long-term culture and are used as attenuated live vaccines to fight tropical theileriosis. Deep microRNA sequencing revealed that infection of both B cells and macrophages alters the expression of a large number of host cell microRNAs. We focused on miR-126-5p as its expression was induced by infection, but diminished in attenuated macrophages that had lost their disease causing disseminating phenotype. We show that miR-126-5p in virulent macrophages directly targets and suppresses a cytosolic scaffold protein called JNK-Interacting Protein-2 (JIP-2), so liberating JNK1 to enter the nucleus and phosphorylate c-Jun. This activates AP-1-driven transcription of *mmp9* that promotes tumour dissemination. In virulent macrophages, an adaptor protein called Grb2 recruits the tyrosine phosphatase PTP1B to AGO2 so decreasing AGO2 phosphorylation to increase miR-126-5p levels. By contrast, in attenuated macrophages AGO2 tyrosine phosphorylation increases and miR-126-5p levels drop leading to a regain in JIP-2 expression that retains JNK1 in the cytosol. This leads to decreased nuclear c-Jun phosphorylation and reduced *mmp9* production. Thus, variations in miR-126-5p levels underpin both virulent hyper-dissemination and attenuation of *T*. *annulata*-transfected macrophages.

## Introduction

*Theileria annulata* is an apicomplexan parasite causing a widespread disease called tropical theileriosis that is endemic to North Africa, the Middle East, vast parts of India and China [1]. The parasite can infect bovine B cells, but in the natural environment, predominantly infects macrophages. *Theileria*-infected leukocytes are transformed into tumour-like leukemias that display uncontrolled proliferation and increased ability to disseminate and invade organs and tissues [2, 3]. As the transformed state of *Theileria*-infected leukocytes can be reversed by drug (buparvaquone)-induced parasite death, molecular events related to *Theileria*-induced host cell transformation have been proposed to have an epigenetic basis [4]. Since attenuated vaccine lines used to fight tropical theileriosis are derived by long-term culture of virulent infected macrophages, and since many virulence traits can be restored by TGF-β2–stimulation of attenuated macrophages [5], loss of *Theileria*-infected macrophage virulence such as activation of the Activator Protein 1 (AP-1) transcription factor [6, 7] may also have an epigenetic element [8, 9].

Micro-ribonucleic acids (miRNAs) are small (17–25 bases long) single-stranded, non-coding RNAs [10, 11] that modulate diverse biological processes by normally binding to the 3′-untranslated region (3’-UTR) of target mRNAs, thus altering the post-transcriptional regulation of numerous genes [12–14]. However, miRNA can also bind to 5'-UTRs, introns and coding sequence of mRNA [15–17]. Post-transcriptional control of gene expression by miRNAs is increasingly recognized as a central part of host/pathogen interactions [18, 19]. The role of miRNAs in bacterial [20, 21], viral [22] and protozoan [23] infections is now well established. A role for miR-155 in the virulence of *T*. *annulata*-infected leukocytes occurs via its suppression of De-Etiolated Homolog 1 (DET-1) expression that diminishes c-Jun ubiquitination [9].

In order to obtain a global view of all bovine miRNAs expressed in different types of *T*. *annulata*-infected leukocytes and how they might contribute to parasite-induced leukocyte transformation and tumour dissemination we determined the miRNomes of infected macrophages (both virulent and attenuated) and infected B cells (TBL20 and TBL3) versus non-infected B cells (BL20 and BL3). Defining the miRNomes of different types of *T*. *annulata*-transformed leukocytes allowed us to observe that infection alters the expression of many known miRNAs. However, to identify miRNAs implicated in *Theileria*-transformed leukocyte dissemination, as opposed to immortalisation, we queried our datasets only for miRNAs whose expression is activated by infection, but dampened upon loss of transformed macrophage dissemination.

Here, we characterise the role of miR-126-5p in *T*. *annulata*-induced leukocyte transformation and attenuation of infected macrophage dissemination. The mixed lineage kinase dual leucine zipper kinase-1 (DLK1) is an established target of miR-126-5p [24]. DLK1 can selectively regulate the JNK-based stress response pathway via its interaction with the scaffolding protein JIP to form a specialized JNK signalling complex [25]. JIP-1 and JIP-2 bind selectively to JNK, but not to other related MAP kinases including p38 [26, 27] and their over-expression causes cytoplasmic retention of JNK; thereby preventing its nuclear translocation and its ability to phosphorylate specific nuclear substrates such as c-Jun [26]. Moreover, under basal conditions DLK1 is bound to JIP, but upon stimulation, DLK1 dissociates from JIP resulting in JNK translocation to the nucleus [28].

Within the nucleus Jun and Fos family members form homo- and hetero-dimers as part of the AP-1 transcription factor that binds to specific DNA sequences to drive target gene expression; a notable example being *mmp9*. The matrix metallo-proteinase MMP9 is associated with metastasis [29] and AP-1 is implicated in various cellular processes such as apoptosis [30], growth control [31] and cellular transformation [32]. Upon activation, JNK translocates to the nucleus to phosphorylate c-Jun that is a key transcription factor in the virulence-associated hyper-dissemination phenotype of *Theileria*-transformed leukocytes [2, 7]. Importantly, upon attenuation of *Theileria*-infected macrophage dissemination JNK activity decreases resulting in reduced c-Jun phosphorylation and decreased AP-1-driven transcription of *mmp9* [33]. We now demonstrate that *T*. *annulata* infection of B cells and macrophages leads to the up-regulation of miR-126-5p that ablates JIP-2 expression liberating cytosolic JNK1 to translocate to the nucleus and phosphorylate c-Jun. Conversely, in attenuated macrophages miR-126-5p levels drop, JIP-2 complexes reform retaining JNK in the cytosol leading to reduced nuclear c-Jun phosphorylation, dampened AP-1-driven transcription of *mmp9* and reduced traversal of matrigel. Thus, high miR-126-5p levels contribute to *Theileria*-transformed leukocyte dissemination and reduced miR-126-5p levels contribute to attenuation of the virulent hyper-dissemination phenotype.

## Results

### Identification of differentially expressed (DE) miRNAs

**Fig 1:**
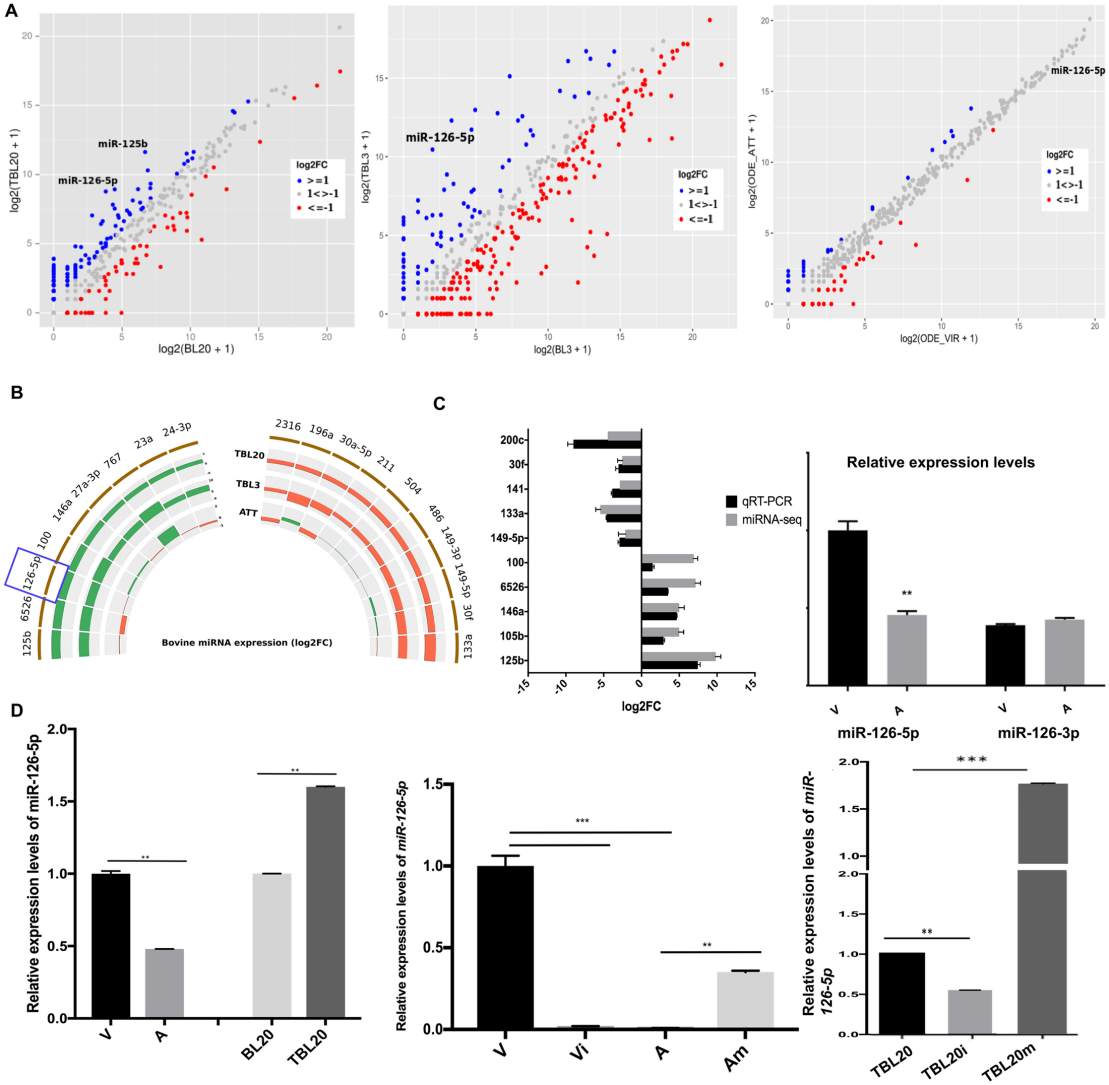
*T*. *annulata* infection modulates the host cells miRNome. **A**. Scatter plot illustrating the log2 fold change of the host cell’s miRNAs in TBL20, TBL3 and attenuated macrophages compared to BL20 and BL3 cells and virulent macrophages. Blue and red dots represent up- and down-regulated miRNAs, respectively (Log2FC>1). **B**. Semi-circular histogram representing the Fold Change values of the common DE miRNAs in TBL20 and TBL3 compared to their uninfected B cells (BL20 and BL3) and their expression in attenuated macrophages versus virulent macrophages. miRNAs are considered differentially expressed (DE) following two criteria: a) fold change (FC) greater than 2 and b) adjusted p value less than 0.05 (DESeq2) and FDR less than 0.1 (baySeq). Orange and green represent down and up-regulated miRNAs, respectively. The miRNA of interest, miR-126-5p, is framed in blue. **C. Left**. qRT-PCR confirmation of the sequencing results in *Theileria*-infected BL20 lymphocytes. **Right**. qRT-PCR confirmation of miR-126-5p and miR-126-3p levels in *Theileria*-infected macrophages. **D. Left**. qRT-PCR confirmation of the relative expression of miR-126-5p in TBL20 compared to BL20 B lymphocytes. **Middle**. qRT-PCR confirmation of the cellular levels of miR-126-5p following transfection of virulent macrophages with inhibitor (Vi) and attenuated macrophages with mimic sequences (Am). **Right**. qRT-PCR confirmation of the cellular levels of miR-126-5p in TBL20 following transfection with mimic (TBL20m) or inhibitor (TBL20i) sequences. The error bars show SD values from 3 biological replicates.

To identify miRNAs whose expression is altered upon infection by *T*. *annulata* we determined the miRNomes of both infected versus non-infected B cells and virulent versus attenuated macrophages. The comparison between *T*. *annulata*-infected TBL20 B lymphocytes and their uninfected counterparts revealed potential involvement of many miRNAs in the transformation of the host cells, as reflected by the changes in their expression levels (Fig 1A). We analyzed the differential expression (DE) of our miRNA sequencing data using DESeq2 [34]. 115 miRNAs are differentially expressed in TBL20 as compared to BL20 with a cutoff of adjusted p value < 0.05. In order to increase the confidence level and limit our analysis to the most significantly DE miRNAs we used a second pipeline, baySeq [35]. Both DESeq2 and baySeq are highly specific and sensitive tools for the detection of differential expression [36]. We consider a miRNA differentially expressed (DE) following two criteria: a) fold change (FC) greater than 2 and b) adjusted p value less than 0.05 (DESeq2) and FDR less than 0.1 (baySeq). Finally, we compared the lists of up- and down-regulated miRNAs from both DESeq2 and baySeq and retained only miRNAs identified as DE in both pipelines (S1 Table).

In addition to TBL20, we characterize the DE miRNAs in a different B cell line: TBL3. Similarly, we identified the DE miRNAs in infected TBL3 as compared to their uninfected counterparts, BL3, using the same pipelines and criteria (S2 Table). The comparison between the lists of DE miRNAs in TBL20 and TBL3 shows that there are 19 common differentially expressed miRNAs: 9 up- and 10 down-regulated. We followed the expression of these 19 DE miRNAs in virulent compared to attenuated macrophages (Fig 1B). This identified miR-126-5p as a miRNA upregulated after *T*. *annulata* infection of B lymphocytes in 2 independent cell lines (TBL20 and TBL3) and down-regulated in attenuated macrophages that have lost their hyper-disseminating phenotype.

As expected for transformed leukocytes the biological functions of the DE miRNAs are annotated as being associated with “oncogenesis”, with the exception of miR-6526 and miR-30f that are not well characterized. The reported functions of the DE miRNAs are therefore consistent with the cancer-like phenotype of *T*. *annulata*-infected leukocytes. To confirm the sequencing results, we randomly selected 10 DE miRNAs, 5 up- and 5 down-regulated and verified their expression qRT-PCR (Fig 1C, left). All tested miRNAs, including miR-126-5p, confirmed the miRNA sequencing data for DE.

#### Validation of miR-126-5p expression levels

miR-126-5p is upregulated upon infection of B cells and in virulent *Theileria*-infected macrophages, and becomes significantly down-regulated upon attenuation of their dissemination capacity (Fig 1C, right and D, left). We overexpressed and inhibited levels of miR-126-5p by transfecting infected leukocytes with miR-126-5p agonists (mimic) and antagonists (inhibitor) (Fig 1D, middle & right). As expected, transfection of *T*. *annulata*-infected TBL20 B cells with antagonist suppressed miR-126-5p expression, where by contrast, transfection with the agonist increased miR-126-5p expression. In macrophages attenuated for dissemination miR-126-5p levels are lower and transfection with the agonist raised miR-126-5p expression, whereas transfection of virulent disseminating macrophages with the antagonist lowered miR-126-5p expression. Clearly, transfection with inhibitor suppresses miR-126-5p expression, and transfection of the mimic greatly increased miR-126-5p expression.

#### Grb2 recruits PTP1B to AGO2 ablating its tyrosine phosphorylation rendering it permissive for miR-126-5p loading

Human miR-126 (which usually refers to the 3′ part of the transcript, also called miR-126-3p) is located within the 7th intron of *EGFL7* gene [37–39]. miR-126-5p refers to the 5′ part of the transcript that is the analogous strand to miR-126-3p, which binds to the main miR-126 transcript in the stem loop structure of the pre-miRNA [38]. Although miR-126-5p and miR-126-3p are derived from the same precursor miRNA, only miR-126-5p and not miR-126-3p targets *DLK1* suggesting they have distinct target-gene specificities [24].

**Fig 2:**
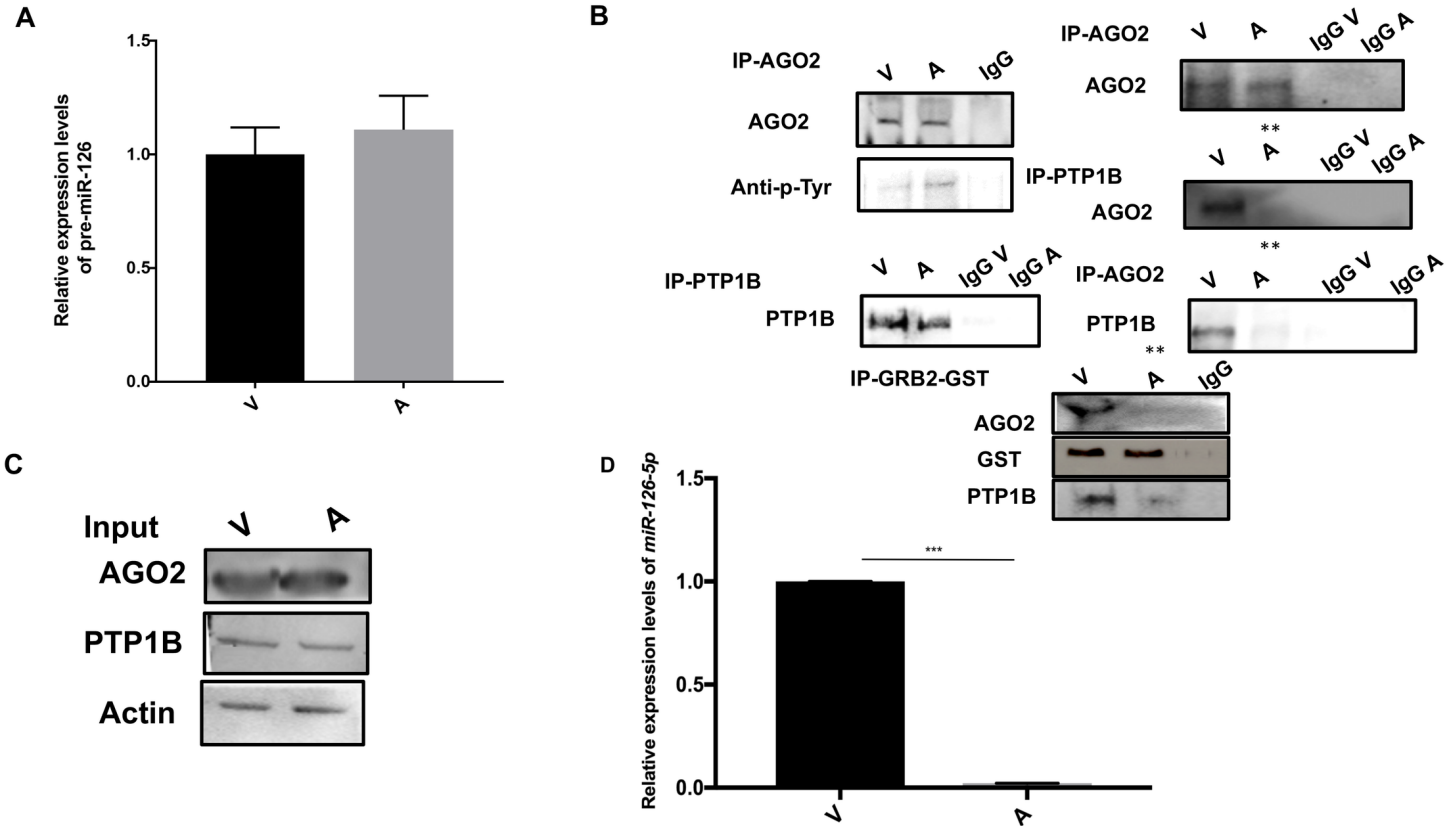
Grb2 recruits PTP1B to AGO2 ablating its tyrosine phosphorylation rendering it permissive for miR-126-5p loading. **A**. Relative expression of pre-miR-126 in virulent (V) and attenuated (A) *Theileria*-infected macrophages. **B**. Immunoprecipitation analyses with anti-AGO2 and anti-PTP1B antibodies using whole cell lysates derived from virulent (V) and attenuated (A) *Theileria*-infected macrophages. **Top** panel shows western blot of the AGO2 precipitate probed with anti-AGO2, anti-phospho-Tyr and PTP1B antibodies. **Middle** panel shows western blot of the PTP1 B precipitate probed with anti-AGO2 and anti-PTP1B antibodies. **Bottom** panel shows pull-down assay performed with GST-Grb2 beads and cell extracts containing endogenous AGO2 expressed by *Theileria*-infected macrophages. Top row shows AGO2 is bound to GST-Grb2 in virulent (V) macrophages, but in attenuated (A) macrophages the small amount of AGO2 bound to Grb2 is below the limit of detection and bottom row shows PTP1B is bound to GST-Grb2 in virulent macrophages. Anti-GST antibodies indicate the amount of Grb2 precipitated. **C**. Western blot analysis of total cell extracts used for immunoprecipitations probed with anti-PTP1B, anti-AGO2 and anti-actin antibodies, the latter used as a loading control. **D**. Relative expression of miR-126-5p in AGO2 precipitates of virulent (V) and attenuated (A) *Theileria*-infected macrophages. All experiments were done independently (n = 3). The error bars show SEM values from 3 biological replicates. ** p value < 0.005; *** p value < 0.001.

*T*. *annulata*-infection of B cells and macrophages results in increased levels of miR-126-5p, while miR-126-3p levels remain low (Fig 1C, right), despite both *EGFL7* (S1 Fig) and pre-miR-126 being equivalently expressed in virulent and attenuated macrophages (Fig 2A). This suggested that only miR-126-5p, and not miR-126-3p, is taken up by AGO2 and protected from degradation [40]. By contrast, in attenuated macrophages miR-126-5p levels drop consistent with it no longer being associated with AGO2. Attenuated macrophages are more oxidatively stressed than virulent macrophages [41] and oxidative stress is known to inhibit PTP1B (Protein Tyrosine Phosphatase 1B) resulting in increased tyrosine phosphorylation of AGO2 and diminished loading of microRNAs [42]. AGO2 was therefore immunoprecipitated from virulent and attenuated macrophages and the phosphorylation status of AGO2 was examined using a phospho-tyrosine specific antibody (Fig 2B, left top panel). Clearly, tyrosine phosphorylation is increased in attenuated macrophages due to reduced amount of PTP1B being associated with AGO2. To confirm this, PTP1B and AGO2 were immunoprecipitated from both virulent and attenuated macrophages and the amount of AGO2 in PTP1B precipitates and PTP1B in AGO2 precipitates examined (Fig 2B, right middle panel). PTP1B was only found associated with AGO2 in virulent macrophages explaining the difficulty to detect it tyrosine phosphorylated. The expression levels of PTP1B and AGO2 are compared to actin in Fig 2C.

We have previously described that the adaptor protein Grb2 recruits p85 regulatory subunit of PI3-K to the TGF-receptor (TGF-R) and that in attenuated macrophages Grb2 levels drop diminishing p85 recruitment to TGF-R [33]. Interestingly, dephosphorylation and deactivation of insulin receptor substrate-1 is facilitated by Grb2 recruitment of PTP1B [43]. This suggested that in virulent macrophages Grb2 might recruit PTP1B to AGO2 resulting in loss of its tyrosine phosphorylation. By contrast, in attenuated macrophages diminished Grb2 expression results in less PTP1B being recruited, so that AGO2 becomes more tyrosine phosphorylated. To verify this GST-Grb2 was used to pull down associated proteins from virulent and attenuated macrophages and the presence of AGO2 and PTP1B detected by western blot (Fig 2B, bottom panel).

In parallel, we immunoprecipitated AGO2 from virulent and attenuated infected macrophages and measured by qRT-PCR the amount of co-precipitated miR-126-5p (Fig 2D). miR-126-5p was readily detected in AGO2 precipitates only from virulent macrophages, where AGO2 is less tyrosine phosphorylated (Fig 2B, left middle panel).

#### Expression levels of miR-126-5p target genes

**Fig 3:**
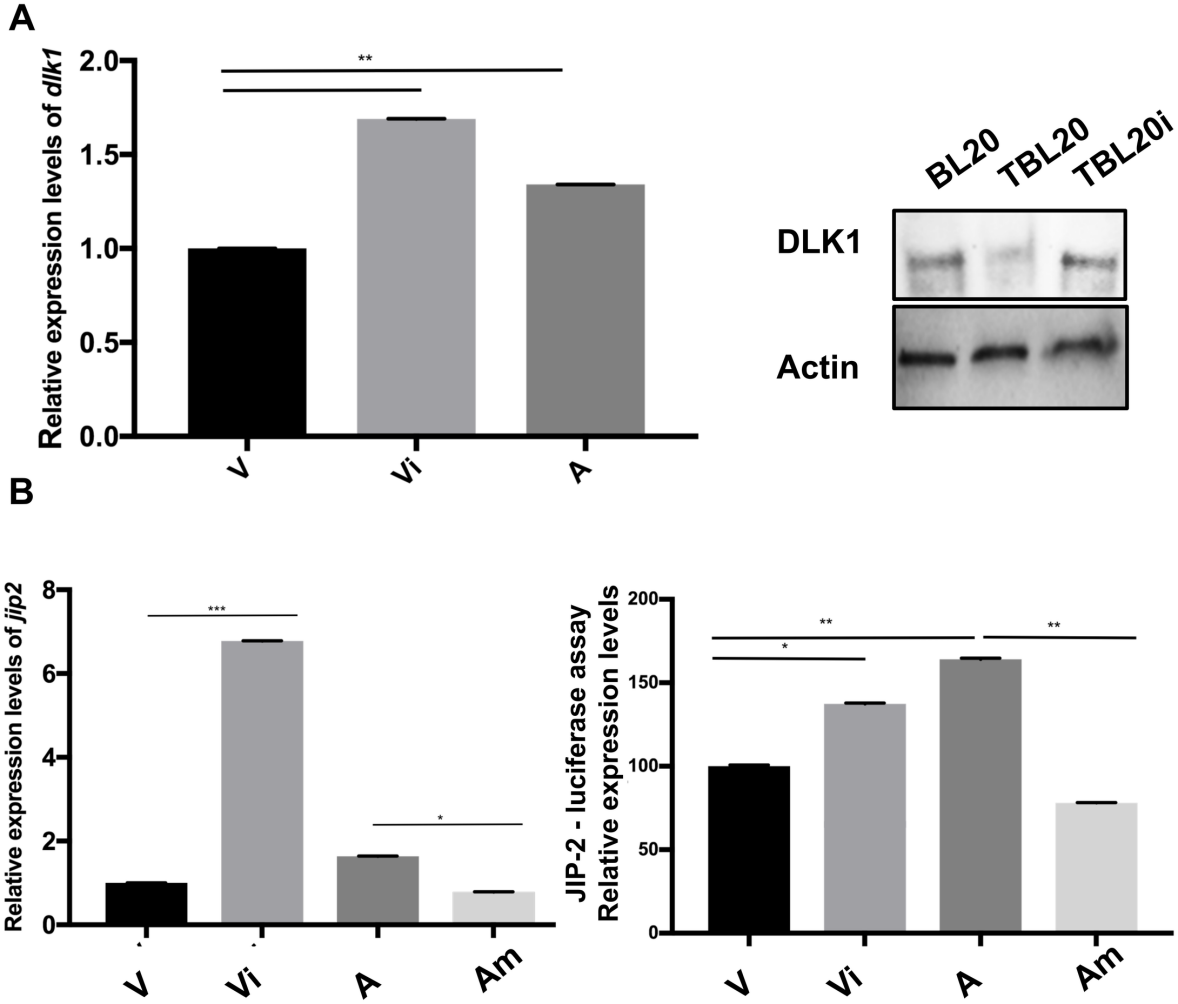
Expression levels of miR-126-5p and its target genes *dlk-1* and *jip-2*. **A**. Expression levels of the established miR-126-5p-target *dlk1* monitored as a positive control. **Left**. Relative expression level of *dlk1* in virulent macrophages (V) compared to virulent macrophages transfected with inhibitor of miR-126 (Vi) and attenuated macrophages (A). **Right**. Protein level of DLK1 in TBL20 cells compared to BL20 cells and TBL20 cells transfected with miR-126-5p inhibitor (TBL20i) **B. Left & right**. Relative expression levels of *JIP-2* in virulent macrophages (V) compared to virulent macrophages transfected with inhibitor of miR-126-5p (Vi) and attenuated macrophages (A) and attenuated macrophages transfected with the mimic of miR-126-5p (Am). The error bars show SEM values from 3 biological replicates. * p value < 0.05, ** p value < 0.01, *** p value < 0.001.

*Dlk1* has been described as a miR-126-5p target gene [24]. Therefore, *Dlk1* transcripts were examined and found reduced in virulent compared to attenuated macrophages (Fig 3A, left) inversely correlating with the higher level of expression of miR-126-5p in virulent macrophages (Fig 1C, right). We then assessed *Dlk1* expression following transfection of virulent macrophages with the miR-126-5p inhibitor sequences. Upon inhibition of miR-126-5p expression the amount of *Dlk1* transcripts increased to above levels observed for attenuated macrophages (Fig 3A, left). Moreover, transfection of TBL20 with the miR-126-5p inhibitor also increased Dlk1 protein levels to those observed for non-infected BL20 B cells (Fig 3A, right). Importantly, JNK Inhibitor Protein (JIP) interacts with Mixed Lineage Kinases (MLKs) including DLK [27]. Since we found higher levels of *Dlk1* transcripts in attenuated macrophages and expression of DLK1 suppressed in TBL20 (Fig 3A) we queried our deep-RNA-seq datasets and found *JIP-2* expression to be upregulated in attenuated macrophages (S1 Fig). This led us to test whether *JIP-2* is a novel direct target gene of miR-126-5p. To this end, transcript levels of *JIP-2* were estimated in virulent and attenuated macrophages transfected with miR-126-5p inhibitor and mimic sequences. Increase in the level of *JIP-2* transcripts occurred upon inhibition of miR-126-5p in virulent macrophages and a decrease in *JIP-2* expression when attenuated macrophages were transfected with the miR-126-5p mimic sequence (Fig 3B, left). Moreover, the 3’-UTR region harboring the identified miR-126-5p seed sequence of bovine *JIP-2* was subcloned into the psiCHECK-2 (Promega, # C8021) and then transfected into *Theileria*-infected macrophages together with the mimic or inhibitor of miR-126-5p and Renilla luciferase activity monitored. An increase in the level of luciferase activity occurred upon inhibition of miR-126-5p in virulent macrophages and a decrease in luciferase activity when miR-126-5p was overexpressed in attenuated macrophages (Fig 3B, right). Taken together, this indicates that *JIP-2* expression is regulated by variations in miR-126-5p levels and confirms that the 3’-UTR of *JIP-2* mRNA possess a bona fide miR-126-5p seed sequence.

#### miR-126-5p expression regulates levels of JIP-2 in *Theileria*-infected macrophages

**Fig 4:**
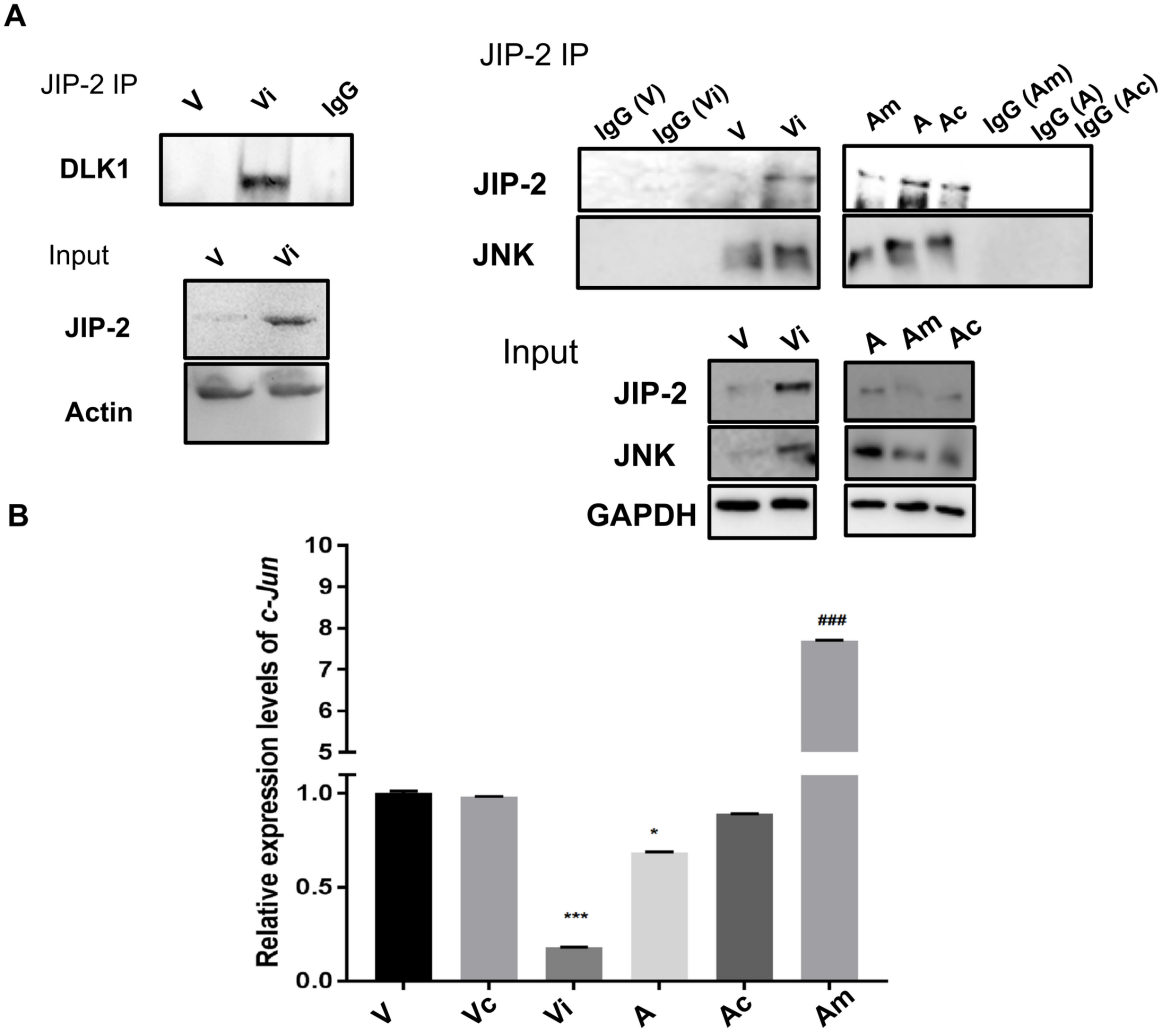
miR-126-5p expression regulates the level of JIP-2 in *Theileria*-infected macrophages. **A**. Immunoprecipitation analyses with anti-JIP-2 antibodies using whole cell lysates derived from virulent (V) *Theileria*-infected macrophages transfected or not with the inhibitor of miR-126-5p (Vi). The top panel (IP-JIP-2) shows western blot of the precipitate probed with an anti-DLK1 antibody, as a positive control. The lower panel shows western blot analysis of total cell extracts used for both immunoprecipitations probed with anti-JIP-2, anti-Dlk1 and anti-actin antibodies, the latter used as a loading control. **B**. Immunoprecipitation analyses with anti-JIP-2 using whole cell lysates derived from virulent (V) and attenuated (A) *Theileria*-infected macrophages transfected or not with a miR-126-5p inhibitor (Vi), mimic (Am) and an irrelevant miR control (Ac). Right and middle upper panel (IP-JIP-2) shows western blot of the precipitate probed with an anti-JIP-2 anti-JNK antibodies. Inhibition of miR-126-5p in virulent macrophages (Vi) increased the formation of the JIP-2/JNK complex, whereas stimulation of miR-126-5p in attenuated macrophages (Am) decreased complex formation. Lower panel shows western blot analysis of total cell extracts used for immunoprecipitations probed with anti-JIP-2, anti-JNK and anti-GAPDH antibodies. In panels A and B IgG represents immunoprecipitation with an irrelevant antibody (IgG). **C**. Relative expression of *c-jun* in virulent and attenuated *Theileria*-infected macrophages before and after transfection with the miR-126-5p inhibitor (Vi), mimic (Am) and an irrelevant control miR (Vc & Ac, NCSTUD002). The error bars show SD values from 3 biological replicates. * p value < 0.05, *** p value < 0.001 and ### p value <0.001 compared to A.

Both JIP-1 and JIP-2 interact with MLKs and facilitate signal transmission by this protein kinase cascade leading to JNK activation, augmented c-Jun phosphorylation and greater AP-1-driven transcription [27]. Due to high miR-126-5p levels JIP-2 is difficult to detect in virulent macrophages, but when they are treated with the miR-126-5p inhibitor JIP-2 is readily observed at levels equivalent to those of attenuated macrophages (Fig 4A and 4B). JIP-2 was immunoprecipitated from inhibitor treated virulent macrophages and DLK1 detected in the precipitate (Fig 4A). Thus, in virulent disseminating macrophages *Theileria*-mediated transformation induces miR-126-5p that represses both JIP-2 and DLK1 expression reducing complex formation below detection levels.

#### miR-126-5p by modulating JIP-2 levels regulates the JNK>c-Jun pathway to sustain AP-1-driven transcription

The cytoplasmic protein JIP binds selectively to JNK [26, 27] and JIP overexpression can act as a powerful inhibitor of JNK signaling [28]. JNK is constitutively activated in *Theileria*-infected leukocytes and its activity downregulated upon attenuation of the hyper-dissemination phenotype [33, 44, 45]. JIP-2 was immunoprecipitated from virulent and attenuated macrophages (Fig 4B, top) and precipitates probed with an anti-JNK antibody (Fig 4B, middle). Due to low levels of JIP-2 in virulent macrophages JNK bound to JIP-2 was more readily detected in attenuated macrophages (Fig 4B, middle). Importantly, inhibition of miR-126-5p in virulent macrophages increased detection of JIP-2-JNK complexes. By contrast, in attenuated macrophages transfected with the miR-126-5p mimic (Am) JIP-2-JNK complexes were difficult to detect (Fig 4B, middle). This demonstrates that miR-126 by modulating JIP-2 expression regulates the amount of JNK bound to JIP-2 in the cytosol.

**Fig 5:**
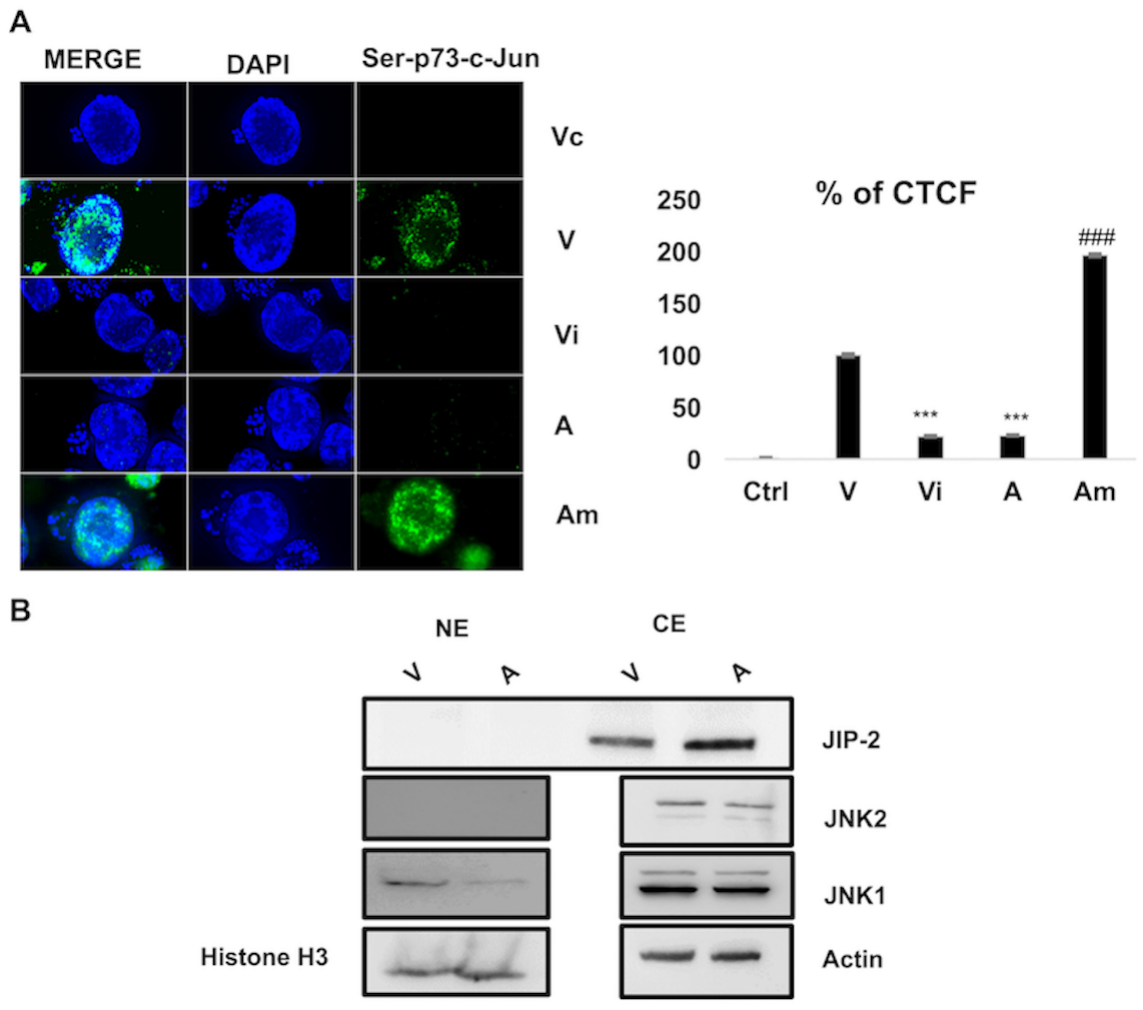
miR-126-5p regulates the JNK phosphorylation of c-Jun by modulating JIP-2 levels. **A. Left**. Immunofluorescence images obtained with anti-phospho-Ser73 c-Jun antibody using virulent (V) and attenuated (A) macrophages transfected or not with the inhibitor (Vi) and mimic of miR-126-5p (Am). Overexpression of miR-126-5p mimic in attenuated macrophages (Am) increased phospho-Ser73-c-Jun staining (green), whereas its inhibition (Vi) in virulent macrophages abolished phospho-Ser73-c-Jun. No fluorescence was observed with Alexa-labelled secondary antibody (Vc). Scale bar is equivalent to 10μ meters. **Right**. Percentage of corrected total cell fluorescence due to phospho-Ser73-c-Jun staining based on 35-independent cell images. **B**. Western blot of JNK isoforms (1 and 2) in nuclear and cytoplasmic extracts. JIP-2-mediated cytosolic retention of JNK1 and decreased therefore nuclear JNK1 in attenuated macrophages. Actin was used as a loading control for cytosolic extracts and histone H3 levels for loading control of nuclear extracts. All experiments were done independently (n = 3). The error bars show SEM values from 3 biological replicates ** p value < 0.01, *** p value < 0.001.

Since miR-126-5p upregulation represses levels of JIP-2 it should facilitate JNK translocation to the nucleus to phosphorylate c-Jun and activate AP-1-driven transcription of target genes such as *c-jun*. Indeed, transfection of miR-126-5p mimic (Am) in attenuated macrophages increased *c-jun* transcript levels, whereas inhibition of miR-126-5p (Vi) in virulent macrophages decreased expression of c-*jun* (Fig 4C). No effect on c-*jun* levels was observed upon transfection of an irrelevant micro-RNA (Ac). Furthermore, in attenuated macrophages ablation of JIP-2 by overexpression of miR-126-5p mimic (Am) increased c-Jun phosphorylation (Fig 5A, left). By contrast, transfection of miR-126-5p inhibitor (Vi) ablates c-Jun phosphorylation in virulent macrophages. No phosphorylation signal is observed when just the Alexa-labeled secondary antibody was used (Vc). Shown (Fig 5, right) is the average Corrected Total Cell Fluorescence (CTCF) for 35 independent cells.

In other cell types JNK1 preferentially phosphorylates c-Jun and in contrast JNK2 decreases c-Jun stability [46]. Since nuclear c-Jun phosphorylation is higher in virulent macrophages compared to attenuated ones, we examined the amount of JNK1 and JNK2 in the nucleus of both virulent and attenuated macrophages. Fig 5B shows the amount of nuclear JNK1 is decreased in attenuated macrophages, where JIP-2 expression is high. JNK2 was undetectable in the nuclear extracts, but readily detectable in cytosolic extracts. Therefore, in attenuated macrophages JIP-2-mediated cytosolic retention of JNK1 leads to decreased nuclear JNK1-mediated c-Jun phosphorylation.

#### miR-126-5p ablation of JIP-2 increases AP-1 transactivation and augments matrigel traversal of transformed leukocytes

**Fig 6:**
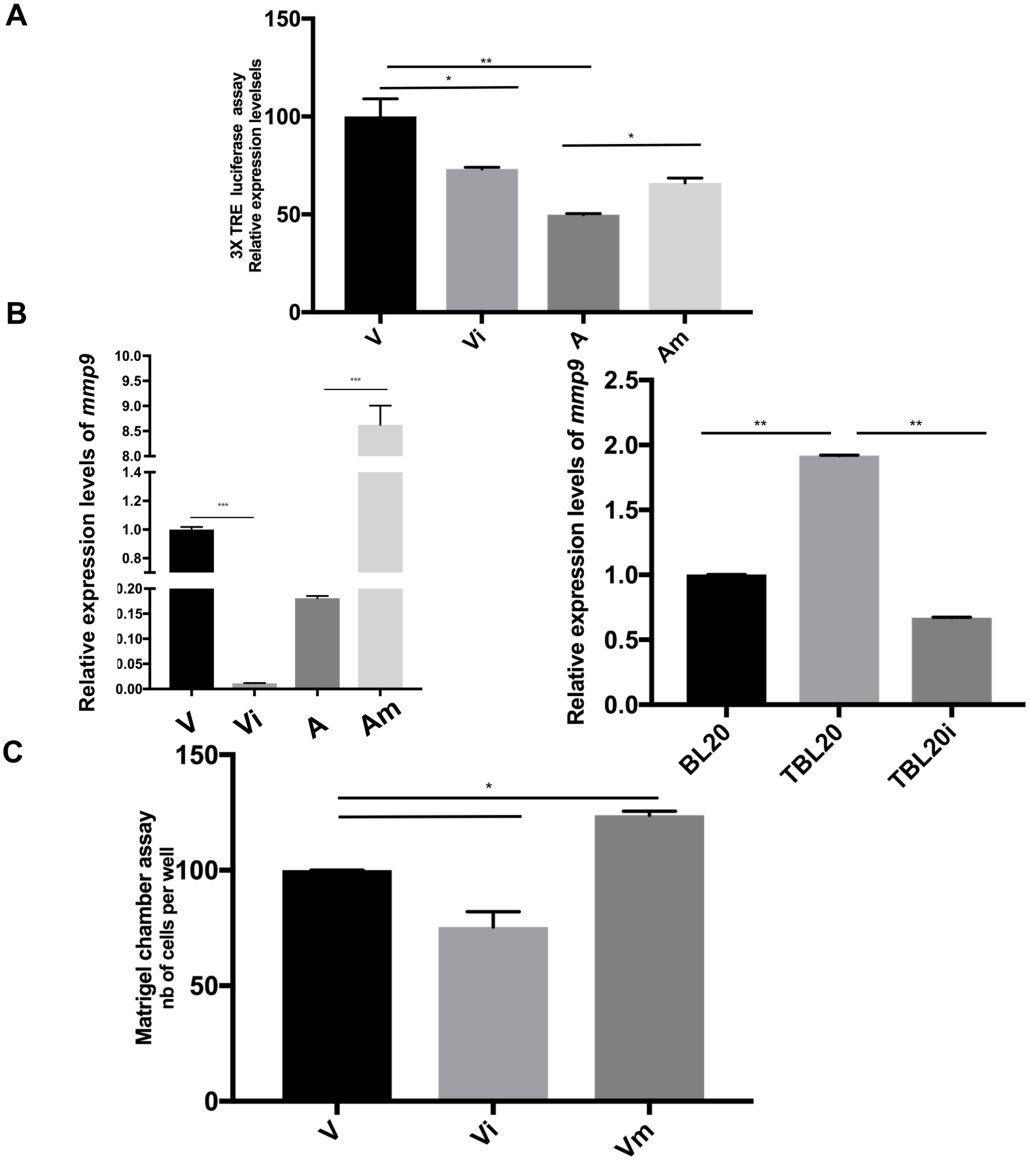
miR-126-5p regulates the JNK1>AP-1 pathway by modulating JIP-2 levels that impact on matrigel traversal of infected leukocytes. **A**. AP-1-(3X-TRE)-driven luciferase activity in virulent macrophages is decreased upon inhibition of miR-126-5p (Vi). When attenuated macrophages are transfected with a miR-126-5p mimic (Am), AP-1-driven luciferase activity increased. **B. Left**. Relative expression of *mmp9* in infected macrophages (V and A) before and after transfection with miR126-5p inhibitor (Vi), mimic (Am) and an irrelevant miRNA control (Vc, NCSTUD002). **Right**. Relative expression of *mmp9* in BL20/TBL20 B cells before and after transfection with miR126-5p inhibitor (TBL20i). **C**. Matrigel traversal of virulent *Theileria*-transformed macrophages. Inhibition of miR-126-5p expression in virulent macrophages (Vi) decreased matrigel traversal, whereas treatment with mimic (Vm) increased matrigel traversal. The reduced traversal capacity of attenuated macrophages is shown (A). All experiments were done independently (n = 3). The error bars show SEM values from 3 biological replicates. * p value < 0.05, ** p value < 0.01, *** p value < 0.001.

As miR-126-5p regulates JIP-2 levels it determines whether JNK1 is retained in the cytosol and consequently it directly impacts on nuclear c-Jun phosphorylation and AP-1 transactivation. Inhibition of miR-126-5p expression increased JIP-2 levels and decreased AP-1-driven transcription in virulent macrophages, whereas the stimulation of miR-126-5p increased AP-1-driven transcription of *mmp9* in attenuated macrophages (Fig 6A and 6B). Thus, changes in miR-126-5p levels alter the degree of AP-1 transactivation in *Theileria*-infected macrophages through modulating JNK1 retention in the cytosol via regulation of JIP-2 levels.

Induction of AP-1 activates transcription of target genes, some of which (e.g. *adam19* and *mmp9*) have been implicated in regulating the dissemination of *Theileria*-infected macrophages [2, 47]. To confirm that miR-126-5p regulates dissemination through modulating AP-1-driven transcription, *mmp9* expression was monitored by RT-PCR (Fig 6B). Upon inhibition of miR-126-5p transcription of *mmp9* decreased in both virulent macrophages and TBL20 B cells (Fig 6B). By contrast, stimulation of miR-126-5p levels raised *mmp9* transcripts in attenuated macrophages (Fig 6B). Clearly, heightened miR-126-5p levels suppress JIP-2 liberating JNK1 to translocate to the nucleus and induce AP-1-driven transcription of *mmp9* to promote the dissemination of virulent *Theileria*-transformed macrophages.

In order to confirm the contribution of miR-126-5p to the dissemination of *Theileria*-transformed leukocytes matrigel chamber assays were performed. As previously described, the capacity of infected virulent macrophages and B cells to traverse matrigel is greater than that of attenuated macrophages and non-infected B cells [2, 6, 8, 47]. However, blockade of miR-126-5p in virulent macrophages diminished their capacity to traverse matrigel, while in contrast, stimulation of miR-126-5p increased matrigel traversal (Fig 6C). The ensemble demonstrates how high miR-126-5p levels in virulent macrophages significantly contribute to their hyper-disseminating phenotype.

## Discussion

Unlike *T*. *parva*, the causative agent of East Coast Fever that results from infection and transformation of T and B cells, *T*. *annulata*-transformed macrophages lose their virulent hyper-disseminating phenotype following long-term culture, and attenuated macrophages with diminished dissemination are used as live vaccines against tropical theileriosis. For these reasons, we used next generation sequencing (NGS) to profile the miRNomes of non-infected B cells, *T*. *annulata*-infected B cells and virulent versus attenuated infected macrophages. The different miRNomes allowed us to compare the miRNA expression of B cells before and following infection and concomitant with loss of *T*. *annulata*-infected macrophage virulence. To focus on miRNAs of particular relevance to parasite-induced leukocyte tumour virulence we asked that expression of a given miRNA be upregulated by infection yet downregulated in attenuated macrophages that have lost their hyper-disseminating phenotype. These criteria identified miR-126-5p as a prime candidate and led to our characterization of its contribution to the transformed phenotype of *T*. *annulata*-infected B cells and macrophages.

**Fig 7:**
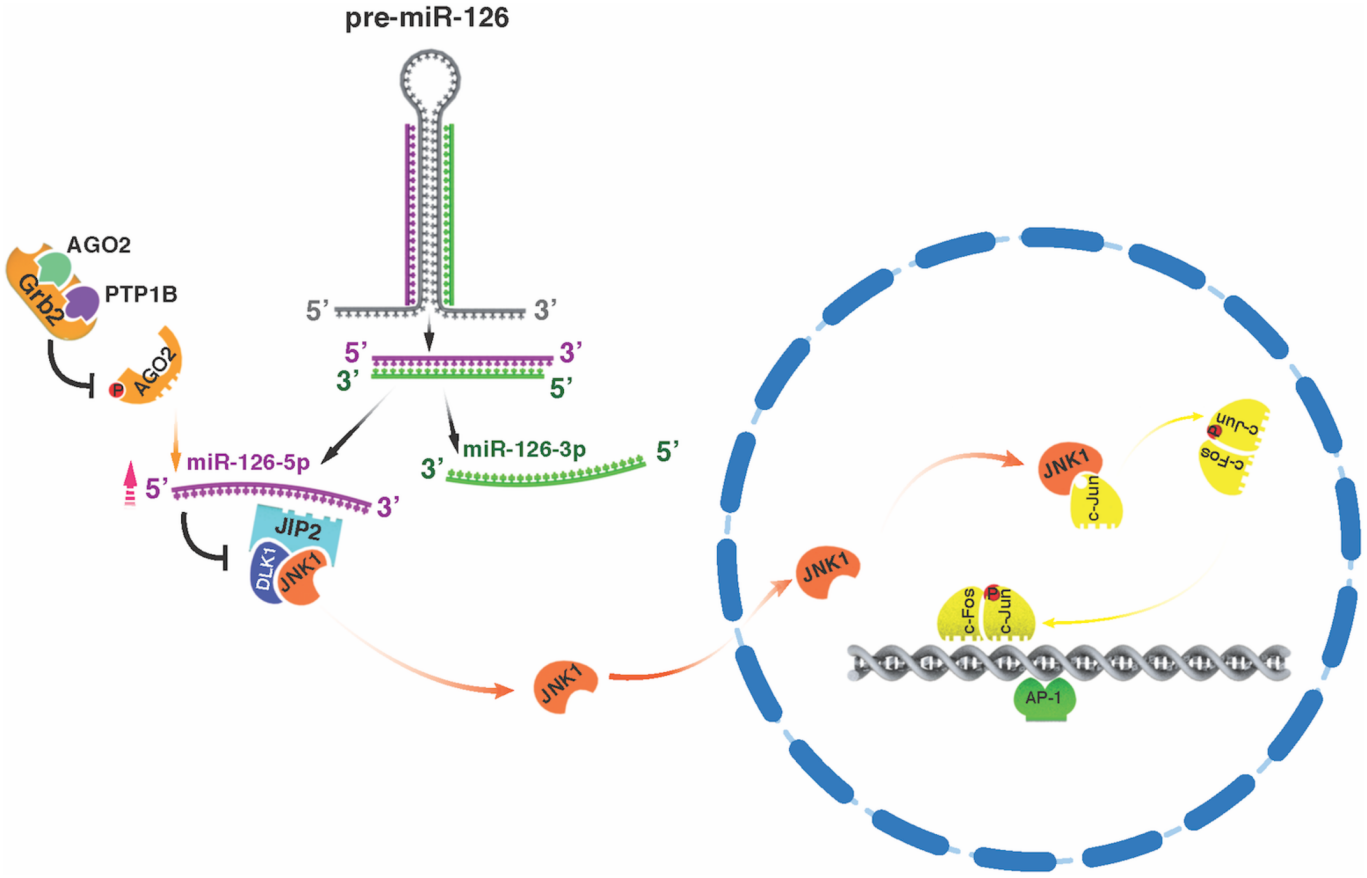
Model proposing how Grb2 recruits PTP1B to AGO2 decreasing its tyrosine phosphorylation leading to loading and protection from degradation of miR-126-5p. Non-degraded miR-126-5p ablates JIP-2 and DLK-1 and releases JNK to translocate to the nucleus and phosphorylate c-Jun. Phospho-c-Jun activates AP-1-driven gene transcription that underpins heightened invasiveness of *Theileria*-transformed virulent macrophages. In this model miR-126-3p is not loaded onto AGO2 and thus is not protected from degradation, explaining the low levels of miR-126-3p detected in virulent and attenuated macrophages.

We demonstrated that *JIP-2* is a novel miR-126-5p target gene and that infection by increasing miR-126-5p levels suppresses JIP-2 expression in virulent macrophages. Loss of JIP-2 released cytosolic JNK1 to translocate to the nucleus and phosphorylate c-Jun, contributing to constitutive AP-1-driven MMP production that is characteristic of *Theileria*-induced leukocyte dissemination. By contrast, in attenuated macrophages, where miR-126-5p expression is reduced, augmented JIP-2 retains JNK1 in the cytosol leading to decreased nuclear c-Jun phosphorylation, ablated MMP9 production and dampened traversal of matrigel. Thus, miR-126-5p-provoked reduction in JIP-2 levels activates JNK1>AP-1 signalling and provides an epigenetic explanation for both *T*. *annulata*-induced leukocyte transformation, and for the attenuated phenotype of live vaccines against tropical theileriosis. By demonstrating that infection-induced miR-126-5p expression ablates JIP-2 and diminishes the cytosolic localisation of JNK1 we provide a mechanism that contributes to constitutive c-Jun phosphorylation, increased MMP9 production and a greater capacity of *Theileria*-transformed leukocytes to disseminate (Fig 7).

miR-126 is located within the 7th intron of the *EGFL7* gene [37–39] and *EGFL7* is equivalently expressed in virulent and attenuated macrophages (S1 Fig). In *T*. *annulata*-infected macrophages miR-126-5p levels therefore do not depend on the degree of *EGFL7* expression [48], nor on the amount of precursor miR-126, rather infection impacts on the capacity of AGO2 to load miR-126-5p, where it’s protected from degradation, while miR-126-3p is not loaded and is consequently degraded. In virulent macrophages Grb2 recruits PTP1B to de-phosphorylate AGO2 that facilitates uptake of miR-126-5p, whereas in attenuated macrophages the amount of PTP1B associated with AGO2 diminishes with a concomitant increase in AGO2 phosphorylation and decrease in bound miR-126-5p (Fig 7). Inflammation stemming from *T*. *annulata* infection likely explains induction of *EGFL7* and pre-miR-126 expression, but why miR-126-5p, rather than miR-126-3p, is loaded onto AGO2 is unknown and will animate future studies. Finally, given that miR-126-5p is deregulated in many cancers; reagents that manipulate miR-126-5p levels could be discussed as tools for cancer therapy.

## Materials and methods

### Cell culture

Cells used in this study are *T*. *annulata*-infected Ode macrophages [49], where virulent macrophages used correspond to passage 62 and attenuated macrophages to passage 364. The different B cell lines used were non-infected immortalized B sarcoma lines (BL3 and BL20) and *T*. *annulata*-infected BL3 (TBL3) and BL20 (TBL20) cells, and all have been previously described [50–53]. All cells were incubated at 37°C with 5% CO_2_ in Roswell Park Memorial Institute medium (RPMI) supplemented with 10% Fetal Bovine Serum (FBS), 2mM L-Glutamine, 100 U penicillin, 0.1mg/ml streptomycin, and 4-(2-hydroxyethyl)-1-piperazineethanesulfonic acid (HEPES) and 5% 2-mercapthoethanol for BL20 and TBL20.

### RNA extractions

Total RNA of *Theileria*-infected leukocytes was isolated using the miRNA isolation kit (#AM1560, Thermo fisher scientific, Villebon, France) according to the manufacturer’s instructions. Total RNA designated for miRNA experiments was extracted using the mirVana miRNA isolation kit (Thermo Fisher) using the manufacturer’s instructions. The quantity and quality checked by Qubit and Bioanalyzer 2100, respectively.

### miRNA library preparation and sequencing

The miRNA libraries were prepared using the Illumina Truseq Small RNA Sample Preparation kit (RS-200-0012) following the manufacturer’s instructions. Briefly, 1 following the manufactupter ligated at the 3’ and 5’ ends, reverse transcribed, barcoded then amplified with 11 cycles of PCR amplifications. Then the cDNA was run on a 6% TBE PAGE gel (Novex, Thermo Fisher). After staining with SYBR Green the gel is visualized on a UV transluminator (Doc-It imaging system, UVP) and the cDNA constructs of a size between 145–160 bp were cut out and eluted from the gel, concentrated and the libraries validated, quantified and finally pooled and sequenced on a Hiseq 2000 and Hiseq 4000.

### miRNA seq data analysis

Raw miRNAseq reads are quality checked by fastqc [54]. mirTools2.0 [55] pipeline is used for trimming (“Adaptor_trim.pl” script) and downstream analysis of known miRNAs. Sequencing reads are aligned to the bosTau7 genome using SOAP [56]. Annotations are added from miRBase 21 [57] and Rfam [58] databases. The differential expression of each known miRNAs from their absolute read counts are analysed by DESeq2 [34]. Differential expression is tested based on the negative Binomial distribution and miRNAs with adjusted p-value < 0.05 considered as statistically significant.

### Plasmid constructs

Bovine genomic DNA of *mapk8* (*jip2*) was purified from *Theileria*-infected macrophages and used as a template for PCR. For construction of the *jip2*, the 3′-UTRs of bovine JIP2 was amplified using the following primers:

Forward: GGCCCTCGAGAGCAGAAAGTTTATTGAGGTGCT

Reverse: GGCCGCGGCCGCTGGGGTCGGAACTGGGAG

The PCR-amplified fragments were digested by *XhoI* and *NotI* and inserted in the psiCHECK-2 vector.

### Measurement of transcriptional activity using Dual-Luciferase reporter assay

Cells were transiently transfected with 2 μg of firefly/renilla luciferase reporter plasmid. Protein extracts were prepared using the Passive Lysis Buffer provided in the Dual-Luciferase Assay (Promega). Equal amounts of protein extracts were plated into a 96-well plate. Firefly luciferase activity was measured for 12 seconds using the LB 960 luminometer (Berthold Technologies, Thoiry, France). To assess the internal standard activity, Stop and Glo reagent was added (Promega), and the peak of the renilla luciferase activity was then measured. Normalized relative luciferase units (RLU) were then calculated as firefly luciferase units of protein extracts of treated or untreated cells divided by renilla luciferase units of protein extracts of untreated cells. Data represent the mean ± SEM of three independent experiments, each performed in duplicate.

### Transfection and AP-1 luciferase assay

Cells were transfected by electroporation using the Nucleofector system (Lonza, Basel, Switzerland). 2.5x10^5^ cells were suspended in 100μl of nucleofector solution mix with 2μg of plasmids and subjected to electroporation using the cell line—specific programme: T-O17. After transfection, cells were suspended in fresh complete medium and incubated at 37°C, 5% CO2 for 24 h and RNA extracted after 48 h post transfections. Measurements of luciferase and β-galactosidase activities were performed using the Dual Light Assay system (Thermo Fisher scientific) and luminometer Centro LB 960 (Berthold) according to the manufacturer’s instructions.

### qRT-PCR of pre-miRNAs

RNA extracted with the mirVana kit was used. cDNA was synthesized using the miScript II RT kit (Qiagen) following the manufacturer’s instructions. Briefly, a 20 μl was set for each biological replicate of each tested miRNA. The RT contained 8 μl MMIX (4 μl of 5x miScript HiFlex Buffer, 2 μl of 10x miScript Nucleics Mix and 2 μl miScript Reverse Transcriptase Mix), 500 ng of total RNA in RNase-free water. The RT was performed at 37°C for 60 min and 95°C for 5 min. The qPCR was performed in a 96-well plate as a 25 μl volume containing 2μl of RT product, 12.5 μl of 2x QuantiTect SYBR Green PCR Master Mix, 2.5 μl of 10x miScript Precursor Assay and RNase-free water. The qPCR thermal cycle was set to 95°C for 15 min and 40 cycles of 94°C for 15 secs, 55°C for 30 secs and 70°C for 30 sec. Data were analysed using the 2−ΔΔCT, or the relative expression method by normalization to HRPT (ENSBTAT00000019547.4) as a reference gene.

### qRT-PCR for miRNAs

RNA extracted with the mirVana kit was used. cDNA was synthesized using the TaqMan microRNA RT kit (Thermo Fisher scientific) following the manufacturer’s instructions. Briefly, a 15 μl was set for each biological replicate of each tested miRNA. The RT contained 7 μl MMIX (100 mM dNTPs, 50 U/μl MultiScribe reverse transcriptase, 10X RT buffer, 20 U/μl RNase inhibitor), 10 ng of total RNA in 5 μl and 3 μl of 5X miRNA-specific RT primers. The RT was performed at 16°C for 30 min, 42°C for 30 min and 85°C for 5 min. The qPCR was performed in a 384well plate as a 10 μl volume containing 1.33 μl of RT product, 5 μl TaqMan 2X universal PCR MMIX, 1 μl of miRNA-specific 20X TaqMan MicroRNA assay. The qPCR thermal cycle was set to 95°C for 10 min and 40 cycles of 95°C for 15 secs and 60°C for 60 sec. Data was analysed using the 2^−ΔΔCT^, or the relative expression method by normalization to U6b as a reference gene for miRNA. bta-miRNA primers were purchased from Thermo Fisher Scientific.

### qRT-PCR of mRNAs

Total RNA of *Theileria*-infected leukocytes was isolated using the RNeasy mini kit (Qiagen), according to the manufacturer’s instructions. The quality and quantity of RNA were measured by Bioanalyzer 2100 and Qubit, respectively. For reverse transcription, 1μg isolated RNA was diluted in water to a final volume of 12 μl, warmed at 65°C for 10 min, then incubated on ice for 2 min. Afterwards, 8 μl of reaction solution (0.5 μl random hexamer, 4 μl 5x RT buffer, 1.5 μl 10mM dNTP, 1 μl 200U/μl RT-MMLV (Promega, Charbonnières-les-Bains, France) and 1 μl 40U/μl RNase inhibitor (Promega) was added to get a final reaction volume of 20 μl and incubated at 37°C for 2 h. The resultant cDNA was stored at -20°C. mRNA expression levels were estimated by qPCR on Light Cycler 480 (Roche, Meylan, France) using SYBR Green detection (Thermo Fisher Scientific). The detection of a single product was verified by dissociation curve analysis and relative quantities of mRNA calculated using the method described by [59].

*gapdh* was used as reference gene to normalize for mRNA levels. The specificity of PCR amplification was confirmed by melting curve analysis. Sequence primers used are as follows:

***gapdh*: FO** 5’-AGGACAAAGCTCAGGGACAC-3’, **Rev** 5’- CCCCAGGTCTACATGTTCCA-3’

***mmp9*: FO** 5’-CCCATTAGCACGCACGACAT-3’, **Rev** 5’- TCACGTAGCCCACATAGTCCA-3’

***dlk1*: FO** 5’- ATGGGCATCGTCTTCCTCAA -3’, **Rev** 5’- CAGGATGGTGAAGCAGATGG -3’

***jip2*: FO** 5’- TCTTCCCTGCCTTCTATGCC -3’, **Rev** 5’- CAGGTGGACGGTCAGTTT -3’

### Western blotting

For total cell extraction, cells were harvested and extracted by lysis buffer (20mM Hepes, Nonidet P40 (NP40) 1%, 0.1% SDS, 150mM NaCl, 2mM EDTA, phosphatase inhibitor cocktail tablet (PhosSTOP, Roche) and protease inhibitor cocktail tablet (Complete mini EDTA free, Roche)). For cytoplasmique extraction, cells were harvested and extracted by lysis buffer (HEPES [10 mM] pH 7.9, KCl [10 mM], EDTA [0.1 mM], NP-40 0.3%, protease inhibitors 1x, protease and phosphatase inhibitor cocktail). For nuclear extraction, cell pellets were lysed and extracted by lysis buffer (HEPES [20 mM] pH 7.9, NaCl [0.4 M], EDTA [1mM], Glycerol 25% and protease Inhibitors 1x.

Protein concentration was determined by the Bradford protein assay [60]. Cell lysates were subjected to Western blot analysis using conventional SDS/PAGE and protein transfer to nitrocellulose filters (Protran, Whatman). The membrane was blocked by 5% non-fat milk-TBST (for anti-DLK, anti-JIP-2, anti-c-JUN, anti-JNK), or 3% non-fat milk-PBST (for anti-actin antibody) for 2 h at room temperature (RT).

Antibodies used in immunoblotting were as follows: goat polyclonal antibody anti-JIP-2 (Santa Cruz Biotechnologies, Heidelberg, Germany # sc-19740), rabbit polyclonal antibody anti-JIP-2 (Abcam # ab-154090), rabbit polyclonal antibody anti-DLK (Santa Cruz Biotechnologies # sc-25437), rabbit polyclonal antibody anti-JNK (Santa Cruz Biotechnologies # sc-571), goat polyclonal anti-PTP1B (Santa Cruz Biotechnologies # sc-1718), mouse monoclonal antibody anti-AGO2 (Abcam # ab-57113), rabbit monoclonal anti-AGO2 (Cell signalling # 2987), mouse anti-phospho tyrosine antibody (Transduction laboratories # P1120), goat anti-GST antibody (GE Healthcare # 27-4577-01) and goat polyclonal antibody anti-actin (Santa Cruz Biotechnologies I-19). After washing, proteins were visualized with ECL western blotting detection reagents (Thermo Scientific). The β-actin level was used as a loading control throughout all experiments.

### Co-immunoprecipitations (Co-IP)

Co-immunoprecipitations and GST-Grb2 pull down assay were conducted with protein extracts of *Theileria*-infected macrophages. JIP-2, AGO2, GST-Grb2 and PTP1B precipitates were transferred to western blots and probed respectively with a rabbit polyclonal anti-DLK, mouse monoclonal anti-AGO2, mouse anti-phospho tyrosine, rabbit anti-PTP1B (Abcam #ab88481) and goat anti-PTP1B antibodies. Normal IgG was used as a negative control, cell lysates from virulent and attenuated macrophages were treated with IgG and the whole cell lysate without IP was included as positive control.

### Immunofluorescence microscopy

1×10^5^ cells were centrifuged on glass slide with the Cellspin I (Tharmac) at 1500 rpm for 10 min and fixed by 4% paraformaldehyde for 10–15 min at room temperature. Fixed cells were permeabilized by 0.2% Triton X-100 for 10 min and blocked with 1% BSA for 30 min. These cells were incubated with primary antibodies against Ser-phospho-73 c-Jun (1/200, Santa Cruz Biotechnologies #sc-7981) overnight, sequentially stained with secondary antibodies conjugated with Alexa 488 (1/1000, Molecular Probes) for 45 min at room temperature. Stained cells were mounted in ProLong Diamond Antifade Mountant with DAPI (Thermo Fisher Scientific). Acquisitions were made by inverted microscopy (Leica DMI6000s) with metamorphous software. Images were taken at x100 magnification.

### Matrigel chambers assay

The invasive capacity of *Theileria*-infected macrophages and B cells were assessed *in vitro* using matrigel migration chambers [7]. Culture coat 96-well medium BME cell invasion assay was obtained from Culturex instructions (3482-096-K). Fifty thousand cells were added to each well and after 24 h of incubation at 37°C, each well of the top chamber was washed once in buffer. The top chamber was placed back on the receiver plate. 100 μl of cell dissociation solution/Calcein AM were added to the bottom chamber of each well, incubated at 37°C for 1 h to fluorescently label cells and dissociate them from the membrane before reading at 485 nm excitation, 520 nm emission using the same parameters as the standard curve.

### Statistical analysis

Data were analysed with the Student’s t-test. All values are expressed as mean+/-SEM. Values were considered to be significantly different when two-sided p values were < 0.05.

### Accession numbers

The miRNA expression dataset has been assigned series record GSE97706 in the GEO repository. The work was conducted under the approval number 15IBEC11 by the Institutional Biosafety and Ethics Committee (IBEC) in KAUST.

## Supporting information

S1 Fig **A**. Relative mRNA expression levels of *egfl7* between virulent (V) and attenuated (A) *Theileria*-infected macrophages. **B**. Relative mRNA expression levels of mapk8ip/JIP-2 between V and A macrophages. The error bars show SD values of 3 biological replicates of the average of Fragments Per Kilobase of transcript per Million mapped reads (FPKM).
(TIF)

S1 Table List of the DE miRNAs in *Theileria*-infected leukocytes (TBL20). (PDF)

S2 Table List of the DE miRNAs in *Theileria*-infected leukocytes (TBL3). (PDF)

## References

[1] Dobbelaere D, Heussler V. Transformation of leukocytes by Theileria parva and T. annulata. Annu Rev Microbiol. 1999;53:1–42. doi: 10.1146/annurev.micro.53.1.1 . 10547684 10.1146/annurev.micro.53.1.1

[2] Echebli N, Mhadhbi M, Chaussepied M, Vayssettes C, Di Santo JP, Darghouth MA, et al Engineering attenuated virulence of a Theileria annulata-infected macrophage. PLoS Negl Trop Dis. 2014;8(11):e3183 doi: 10.1371/journal.pntd.0003183 25375322 10.1371/journal.pntd.0003183 PMC4222746

[3] Somerville RP, Adamson RE, Brown CG, Hall FR. Metastasis of Theileria annulata macroschizont-infected cells in scid mice is mediated by matrix metalloproteinases. Parasitology. 1998;116 (Pt 3):223–8. . 9550215 10.1017/s0031182097002151

[4] Cheeseman K, Weitzman JB. Host-parasite interactions: an intimate epigenetic relationship. Cell Microbiol. 2015;17(8):1121–32. doi: 10.1111/cmi.12471 . 26096716 10.1111/cmi.12471

[5] Chaussepied M, Janski N, Baumgartner M, Lizundia R, Jensen K, Weir W, et al TGF-b2 induction regulates invasiveness of Theileria-transformed leukocytes and disease susceptibility. PLoS Pathog. 2010;6(11):e1001197 doi: 10.1371/journal.ppat.1001197 . 21124992 10.1371/journal.ppat.1001197 PMC2987823

[6] Baylis HA, Megson A, Hall R. Infection with Theileria annulata induces expression of matrix metalloproteinase 9 and transcription factor AP-1 in bovine leucocytes. Mol Biochem Parasitol. 1995;69(2):211–22. . 7770085 10.1016/0166-6851(94)00216-a

[7] Lizundia R, Chaussepied M, Huerre M, Werling D, Di Santo JP, Langsley G. c-Jun NH2-terminal kinase/c-Jun signaling promotes survival and metastasis of B lymphocytes transformed by Theileria. Cancer Res. 2006;66(12):6105–10. doi: 10.1158/0008-5472.CAN-05-3861 . 16778183 10.1158/0008-5472.CAN-05-3861

[8] Cock-Rada AM, Medjkane S, Janski N, Yousfi N, Perichon M, Chaussepied M, et al SMYD3 promotes cancer invasion by epigenetic upregulation of the metalloproteinase MMP-9. Cancer Res. 2012;72(3):810–20. doi: 10.1158/0008-5472.CAN-11-1052 22194464 10.1158/0008-5472.CAN-11-1052 PMC3299564

[9] Marsolier J, Pineau S, Medjkane S, Perichon M, Yin Q, Flemington E, et al OncomiR addiction is generated by a miR-155 feedback loop in Theileria-transformed leukocytes. PLoS Pathog. 2013;9(4):e1003222 doi: 10.1371/journal.ppat.1003222 23637592 10.1371/journal.ppat.1003222 PMC3630095

[10] Aslam MI, Taylor K, Pringle JH, Jameson JS. MicroRNAs are novel biomarkers of colorectal cancer. Br J Surg. 2009;96(7):702–10. doi: 10.1002/bjs.6628 . 19526617 10.1002/bjs.6628

[11] El-Murr N, Abidi Z, Wanherdrick K, Svrcek M, Gaub MP, Flejou JF, et al MiRNA genes constitute new targets for microsatellite instability in colorectal cancer. PLoS One. 2012;7(2):e31862 doi: 10.1371/journal.pone.0031862 22348132 10.1371/journal.pone.0031862 PMC3279428

[12] Delay C, Calon F, Mathews P, Hebert SS. Alzheimer-specific variants in the 3'UTR of Amyloid precursor protein affect microRNA function. Mol Neurodegener. 2011;6:70 doi: 10.1186/1750-1326-6-70 21982160 10.1186/1750-1326-6-70 PMC3195754

[13] Kruger J, Rehmsmeier M. RNAhybrid: microRNA target prediction easy, fast and flexible. Nucleic Acids Res. 2006;34(Web Server issue):W451–4. doi: 10.1093/nar/gkl243 16845047 10.1093/nar/gkl243 PMC1538877

[14] Satoh M, Minami Y, Takahashi Y, Tabuchi T, Nakamura M. A cellular microRNA, let-7i, is a novel biomarker for clinical outcome in patients with dilated cardiomyopathy. J Card Fail. 2011;17(11):923–9. doi: 10.1016/j.cardfail.2011.07.012 . 22041329 10.1016/j.cardfail.2011.07.012

[15] Moretti F, Thermann R, Hentze MW. Mechanism of translational regulation by miR-2 from sites in the 5' untranslated region or the open reading frame. RNA. 2010;16(12):2493–502. doi: 10.1261/rna.2384610 20966199 10.1261/rna.2384610 PMC2995410

[16] Kulkarni S, Savan R, Qi Y, Gao X, Yuki Y, Bass SE, et al Differential microRNA regulation of HLA-C expression and its association with HIV control. Nature. 2011;472(7344):495–8. doi: 10.1038/nature09914 21499264 10.1038/nature09914 PMC3084326

[17] Duursma AM, Kedde M, Schrier M, le Sage C, Agami R. miR-148 targets human DNMT3b protein coding region. RNA. 2008;14(5):872–7. doi: 10.1261/rna.972008 18367714 10.1261/rna.972008 PMC2327368

[18] Hakimi MA, Deitsch KW. Epigenetics in Apicomplexa: control of gene expression during cell cycle progression, differentiation and antigenic variation. Curr Opin Microbiol. 2007;10(4):357–62. doi: 10.1016/j.mib.2007.07.005 . 17719264 10.1016/j.mib.2007.07.005

[19] Judice CC, Bourgard C, Kayano AC, Albrecht L, Costa FT. MicroRNAs in the Host-Apicomplexan Parasites Interactions: A Review of Immunopathological Aspects. Front Cell Infect Microbiol. 2016;6:5 doi: 10.3389/fcimb.2016.00005 26870701 10.3389/fcimb.2016.00005 PMC4735398

[20] Eulalio A, Schulte L, Vogel J. The mammalian microRNA response to bacterial infections. RNA Biol. 2012;9(6):742–50. doi: 10.4161/rna.20018 . 22664920 10.4161/rna.20018

[21] Staedel C, Darfeuille F. MicroRNAs and bacterial infection. Cell Microbiol. 2013;15(9):1496–507. doi: 10.1111/cmi.12159 . 23795564 10.1111/cmi.12159

[22] Cullen BR. Viruses and microRNAs: RISCy interactions with serious consequences. Genes Dev. 2011;25(18):1881–94. doi: 10.1101/gad.17352611 21896651 10.1101/gad.17352611 PMC3185961

[23] LaMonte G, Philip N, Reardon J, Lacsina JR, Majoros W, Chapman L, et al Translocation of sickle cell erythrocyte microRNAs into Plasmodium falciparum inhibits parasite translation and contributes to malaria resistance. Cell Host Microbe. 2012;12(2):187–99. doi: 10.1016/j.chom.2012.06.007 22901539 10.1016/j.chom.2012.06.007 PMC3442262

[24] Schober A, Nazari-Jahantigh M, Wei Y, Bidzhekov K, Gremse F, Grommes J, et al MicroRNA-126-5p promotes endothelial proliferation and limits atherosclerosis by suppressing Dlk1. Nat Med. 2014;20(4):368–76. doi: 10.1038/nm.3487 24584117 10.1038/nm.3487 PMC4398028

[25] Whitmarsh AJ. The JIP family of MAPK scaffold proteins. Biochem Soc Trans. 2006;34(Pt 5):828–32. doi: 10.1042/BST0340828 . 17052208 10.1042/BST0340828

[26] Dickens M, Rogers JS, Cavanagh J, Raitano A, Xia Z, Halpern JR, et al A cytoplasmic inhibitor of the JNK signal transduction pathway. Science. 1997;277(5326):693–6. . 9235893 10.1126/science.277.5326.693

[27] Yasuda J, Whitmarsh AJ, Cavanagh J, Sharma M, Davis RJ. The JIP group of mitogen-activated protein kinase scaffold proteins. Mol Cell Biol. 1999;19(10):7245–54. 10490659 10.1128/mcb.19.10.7245 PMC84717

[28] Nihalani D, Wong HN, Holzman LB. Recruitment of JNK to JIP1 and JNK-dependent JIP1 phosphorylation regulates JNK module dynamics and activation. J Biol Chem. 2003;278(31):28694–702. doi: 10.1074/jbc.M304212200 . 12756254 10.1074/jbc.M304212200

[29] Stetler-Stevenson WG, Aznavoorian S, Liotta LA. Tumor cell interactions with the extracellular matrix during invasion and metastasis. Annu Rev Cell Biol. 1993;9:541–73. doi: 10.1146/annurev.cb.09.110193.002545 . 8280471 10.1146/annurev.cb.09.110193.002545

[30] Bossy-Wetzel E, Bakiri L, Yaniv M. Induction of apoptosis by the transcription factor c-Jun. EMBO J. 1997;16(7):1695–709. doi: 10.1093/emboj/16.7.1695 9130714 10.1093/emboj/16.7.1695 PMC1169773

[31] Kovary K, Bravo R. Existence of different Fos/Jun complexes during the G0-to-G1 transition and during exponential growth in mouse fibroblasts: differential role of Fos proteins. Mol Cell Biol. 1992;12(11):5015–23. 1406676 10.1128/mcb.12.11.5015-5023.1992 PMC360434

[32] Vandel L, Montreau N, Vial E, Pfarr CM, Binetruy B, Castellazzi M. Stepwise transformation of rat embryo fibroblasts: c-Jun, JunB, or JunD can cooperate with Ras for focus formation, but a c-Jun-containing heterodimer is required for immortalization. Mol Cell Biol. 1996;16(5):1881–8. 8628254 10.1128/mcb.16.5.1881 PMC231175

[33] Haidar M, Whitworth J, Noe G, Liu WQ, Vidal M, Langsley G. TGF-beta2 induces Grb2 to recruit PI3-K to TGF-RII that activates JNK/AP-1-signaling and augments invasiveness of Theileria-transformed macrophages. Sci Rep. 2015;5:15688 doi: 10.1038/srep15688 26511382 10.1038/srep15688 PMC4625156

[34] Love MI, Huber W, Anders S. Moderated estimation of fold change and dispersion for RNA-seq data with DESeq2. Genome Biol. 2014;15(12):550 doi: 10.1186/s13059-014-0550-8 25516281 10.1186/s13059-014-0550-8 PMC4302049

[35] Hardcastle TJ, Kelly KA. baySeq: empirical Bayesian methods for identifying differential expression in sequence count data. BMC Bioinformatics. 2010;11:422 doi: 10.1186/1471-2105-11-422 20698981 10.1186/1471-2105-11-422 PMC2928208

[36] Cordero F, Beccuti M, Arigoni M, Donatelli S, Calogero RA. Optimizing a massive parallel sequencing workflow for quantitative miRNA expression analysis. PLoS One. 2012;7(2):e31630 doi: 10.1371/journal.pone.0031630 22363693 10.1371/journal.pone.0031630 PMC3282730

[37] Fish JE, Santoro MM, Morton SU, Yu S, Yeh RF, Wythe JD, et al miR-126 regulates angiogenic signaling and vascular integrity. Dev Cell. 2008;15(2):272–84. doi: 10.1016/j.devcel.2008.07.008 18694566 10.1016/j.devcel.2008.07.008 PMC2604134

[38] Meister J, Schmidt MH. miR-126 and miR-126*: new players in cancer. ScientificWorldJournal. 2010;10:2090–100. doi: 10.1100/tsw.2010.198 . 20953557 10.1100/tsw.2010.198 PMC5763667

[39] Wang S, Aurora AB, Johnson BA, Qi X, McAnally J, Hill JA, et al The endothelial-specific microRNA miR-126 governs vascular integrity and angiogenesis. Dev Cell. 2008;15(2):261–71. doi: 10.1016/j.devcel.2008.07.002 18694565 10.1016/j.devcel.2008.07.002 PMC2685763

[40] Schmitter D, Filkowski J, Sewer A, Pillai RS, Oakeley EJ, Zavolan M, et al Effects of Dicer and Argonaute down-regulation on mRNA levels in human HEK293 cells. Nucleic Acids Res. 2006;34(17):4801–15. doi: 10.1093/nar/gkl646 16971455 10.1093/nar/gkl646 PMC1635286

[41] Metheni M, Echebli N, Chaussepied M, Ransy C, Chereau C, Jensen K, et al The level of H(2)O(2) type oxidative stress regulates virulence of Theileria-transformed leukocytes. Cell Microbiol. 2014;16(2):269–79. doi: 10.1111/cmi.12218 24112286 10.1111/cmi.12218 PMC3906831

[42] Yang M, Haase AD, Huang FK, Coulis G, Rivera KD, Dickinson BC, et al Dephosphorylation of tyrosine 393 in argonaute 2 by protein tyrosine phosphatase 1B regulates gene silencing in oncogenic RAS-induced senescence. Mol Cell. 2014;55(5):782–90. doi: 10.1016/j.molcel.2014.07.018 25175024 10.1016/j.molcel.2014.07.018 PMC4159145

[43] Goldstein BJ, Bittner-Kowalczyk A, White MF, Harbeck M. Tyrosine dephosphorylation and deactivation of insulin receptor substrate-1 by protein-tyrosine phosphatase 1B. Possible facilitation by the formation of a ternary complex with the Grb2 adaptor protein. J Biol Chem. 2000;275(6):4283–9. . 10660596 10.1074/jbc.275.6.4283

[44] Chaussepied M, Lallemand D, Moreau MF, Adamson R, Hall R, Langsley G. Upregulation of Jun and Fos family members and permanent JNK activity lead to constitutive AP-1 activation in Theileria-transformed leukocytes. Mol Biochem Parasitol. 1998;94(2):215–26. . 9747972 10.1016/s0166-6851(98)00070-x

[45] Galley Y, Hagens G, Glaser I, Davis W, Eichhorn M, Dobbelaere D. Jun NH2-terminal kinase is constitutively activated in T cells transformed by the intracellular parasite Theileria parva. Proc Natl Acad Sci U S A. 1997;94(10):5119–24. 9144200 10.1073/pnas.94.10.5119 PMC24641

[46] Bode AM, Dong Z. The functional contrariety of JNK. Mol Carcinog. 2007;46(8):591–8. doi: 10.1002/mc.20348 17538955 10.1002/mc.20348 PMC2832829

[47] Adamson R, Logan M, Kinnaird J, Langsley G, Hall R. Loss of matrix metalloproteinase 9 activity in Theileria annulata-attenuated cells is at the transcriptional level and is associated with differentially expressed AP-1 species. Mol Biochem Parasitol. 2000;106(1):51–61. . 10743610 10.1016/s0166-6851(99)00213-3

[48] Monteys AM, Spengler RM, Wan J, Tecedor L, Lennox KA, Xing Y, et al Structure and activity of putative intronic miRNA promoters. RNA. 2010;16(3):495–505. doi: 10.1261/rna.1731910 20075166 10.1261/rna.1731910 PMC2822915

[49] Singh S, Khatri N, Manuja A, Sharma RD, Malhotra DV, Nichani AK. Impact of field vaccination with a Theileria annulata schizont cell culture vaccine on the epidemiology of tropical theileriosis. Vet Parasitol. 2001;101(2):91–100. . 11587838 10.1016/s0304-4017(01)00502-7

[50] Morzaria S, Roeder P, Roberts D, Chasey D, Drew T. Characteristics of a continuous suspension cell line derived from a calf with sporadic leukosis. In: Straub OC, editor. 1982:519–28.

[51] Shiels BR, McDougall C, Tait A, Brown CG. Identification of infection-associated antigens in Theileria annulata transformed cells. Parasite Immunol. 1986;8(1):69–77. . 2421227 10.1111/j.1365-3024.1986.tb00834.x

[52] Moreau MF, Thibaud JL, Miled LB, Chaussepied M, Baumgartner M, Davis WC, et al Theileria annulata in CD5(+) macrophages and B1 B cells. Infect Immun. 1999;67(12):6678–82. 10569790 10.1128/iai.67.12.6678-6682.1999 PMC97082

[53] Theilen GH, Rush JD, Nelson-Rees WA, Dungworth DL, Munn RJ, Switzer JW. Bovine leukemia: establishment and morphologic characterization of continuous cell suspension culture, BL-1. J Natl Cancer Inst. 1968;40(4):737–49. . 4171552

[54] Andrews S. FastQC: a quality control tool for high throughput sequence data. 2010: http://www.bioinformatics.babraham.ac.uk/projects/fastqc.

[55] Wu J, Liu Q, Wang X, Zheng J, Wang T, You M, et al mirTools 2.0 for non-coding RNA discovery, profiling, and functional annotation based on high-throughput sequencing. RNA Biol. 2013;10(7):1087–92. doi: 10.4161/rna.25193 23778453 10.4161/rna.25193 PMC3849156

[56] Li R, Li Y, Kristiansen K, Wang J. SOAP: short oligonucleotide alignment program. Bioinformatics. 2008;24(5):713–4. doi: 10.1093/bioinformatics/btn025 . 18227114 10.1093/bioinformatics/btn025

[57] Kozomara A, Griffiths-Jones S. miRBase: annotating high confidence microRNAs using deep sequencing data. Nucleic Acids Res. 2014;42(Database issue):D68–73. doi: 10.1093/nar/gkt1181 24275495 10.1093/nar/gkt1181 PMC3965103

[58] Nawrocki EP, Burge SW, Bateman A, Daub J, Eberhardt RY, Eddy SR, et al Rfam 12.0: updates to the RNA families database. Nucleic Acids Res. 2015;43(Database issue):D130–7. doi: 10.1093/nar/gku1063 25392425 10.1093/nar/gku1063 PMC4383904

[59] Pfaffl MW. A new mathematical model for relative quantification in real-time RT-PCR. Nucleic Acids Res. 2001;29(9):e45 11328886 10.1093/nar/29.9.e45 PMC55695

[60] Okutucu B, Dincer A, Habib O, Zihnioglu F. Comparison of five methods for determination of total plasma protein concentration. J Biochem Biophys Methods. 2007;70(5):709–11. doi: 10.1016/j.jbbm.2007.05.009 . 17597224 10.1016/j.jbbm.2007.05.009

