# Supplementary figures and images for: miR-126-5p by direct targeting of JNK-interacting protein-2 (JIP-2) plays a key role in Theileria-infected macrophage virulence
Source: PLoS Pathog. 2018 Mar 23;14(3):e1006942. doi: 10.1371/journal.ppat.1006942 (PMC5892942; doi:10.1371/journal.ppat.1006942)

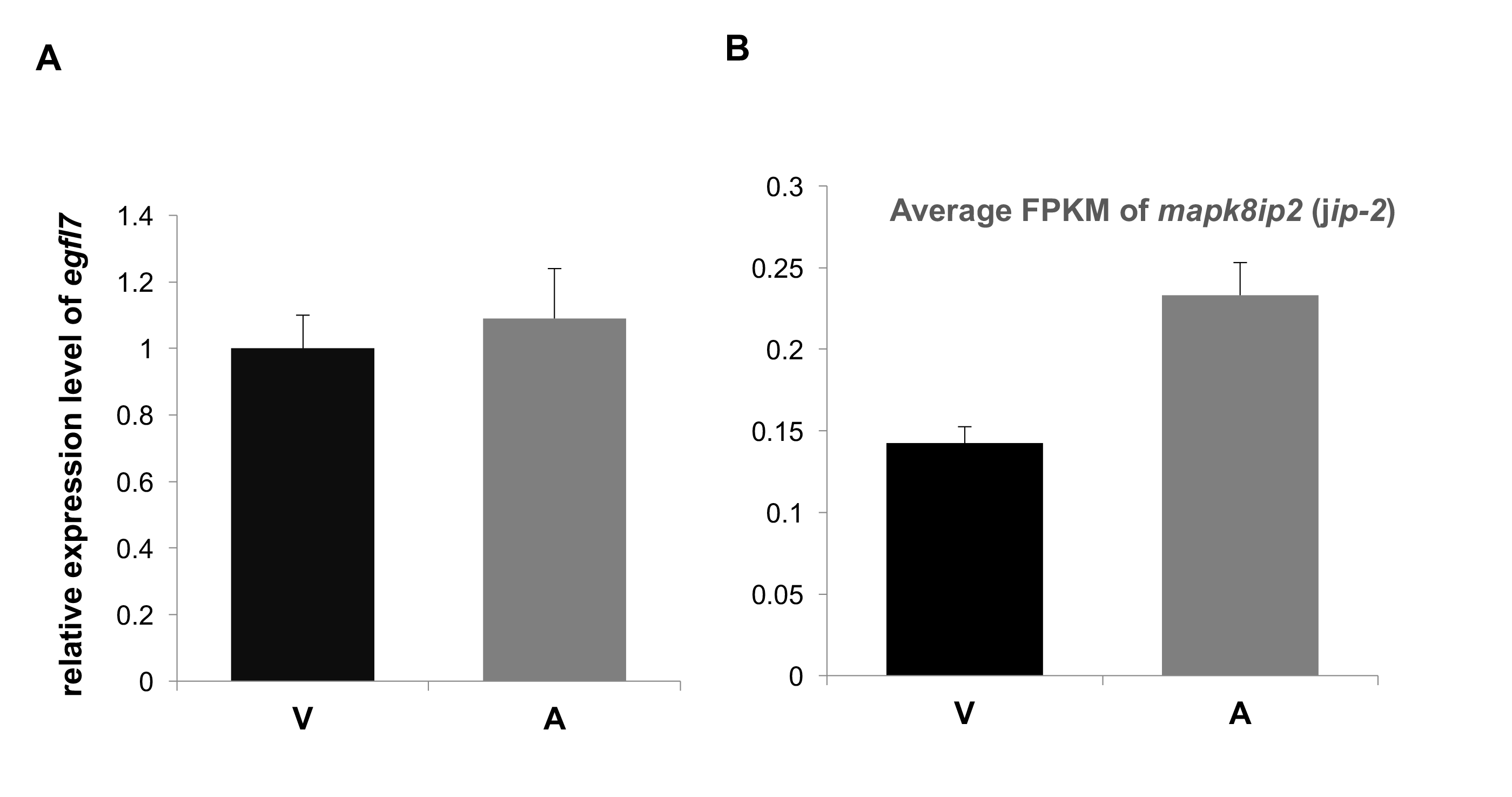

Supplement: S1 Fig — A. Relative mRNA expression levels of egfl7 between virulent (V) and attenuated (A) Theileria-infected macrophages. B. Relative mRNA expression levels of mapk8ip/JIP-2 between V and A macrophages. The error bars show SD values of 3 biological replicates of the average of Fragments Per Kilobase of transcript per Million mapped reads (FPKM). (TIF) [file ppat.1006942.s001.tif]
