# Supplementary material for: miR-126-5p by direct targeting of JNK-interacting protein-2 (JIP-2) plays a key role in Theileria-infected macrophage virulence
Source: PLoS Pathog. 2018 Mar 23;14(3):e1006942. doi: 10.1371/journal.ppat.1006942 (PMC5892942; doi:10.1371/journal.ppat.1006942)
Supplement: S1 Table — (PDF) [file ppat.1006942.s002.pdf]

Column1	log2FoldChange	pvalue	padj
bta-miR-125b	4.735	1.68E-10	2.37E-09
bta-miR-6526	4.525	5.45E-26	2.65E-24
bta-miR-105b	4.331	2.26E-19	5.82E-18
bta-miR-126-5p	4.305	7.24E-31	4.53E-29
bta-miR-2400	4.087	8.52E-05	7.04E-04
bta-miR-126-3p	3.939	1.85E-04	1.42E-03
bta-miR-100	3.787	4.19E-20	1.15E-18
bta-miR-2425-5p	3.494	4.47E-05	3.84E-04
bta-miR-145	3.298	6.63E-04	4.27E-03
bta-miR-146a	3.115	3.37E-44	3.69E-42
bta-miR-205	3.090	2.01E-03	1.03E-02
bta-miR-1298	3.015	7.01E-03	3.04E-02
bta-miR-138	2.865	7.21E-19	1.75E-17
bta-miR-105a	2.834	1.87E-11	3.27E-10
bta-miR-2461-3p	2.707	1.21E-03	6.89E-03
bta-miR-199a-3p	2.589	6.55E-03	2.87E-02
bta-miR-27a-3p	2.585	4.00E-24	1.59E-22
bta-miR-767	2.558	3.08E-26	1.69E-24
bta-miR-23a	2.552	3.85E-25	1.68E-23
bta-miR-27a-5p	2.510	4.41E-11	6.90E-10
bta-miR-143	2.451	8.48E-14	1.61E-12
bta-miR-24-3p	2.022	2.22E-11	3.74E-10
bta-miR-34c	1.836	2.47E-08	3.00E-07
bta-miR-148a	1.689	6.03E-17	1.39E-15
bta-miR-1271	-1.950	3.10E-03	1.51E-02
bta-miR-2316	-2.087	5.92E-09	7.63E-08
bta-miR-2474	-2.119	9.32E-23	3.14E-21
bta-miR-196a	-2.354	2.02E-23	7.37E-22
bta-let-7b	-2.359	5.18E-08	6.14E-07
bta-miR-30a-5p	-2.373	1.56E-22	4.55E-21
bta-miR-211	-2.422	3.39E-04	2.36E-03
bta-miR-320a	-2.620	3.03E-03	1.49E-02
bta-miR-181c	-2.649	7.85E-04	4.71E-03
bta-miR-204	-2.811	1.68E-03	8.75E-03
bta-miR-504	-2.922	7.60E-13	1.39E-11
bta-miR-486	-2.931	2.75E-201	1.21E-198
bta-miR-127	-2.962	3.86E-16	8.45E-15
bta-miR-149-3p	-3.015	6.88E-07	7.72E-06
bta-miR-615	-3.078	1.36E-03	7.42E-03
bta-miR-149-5p	-3.133	2.25E-34	1.64E-32
bta-miR-154c	-3.188	5.22E-03	2.41E-02
bta-miR-485	-3.519	1.67E-03	8.75E-03
bta-miR-2394	-3.589	6.08E-04	3.98E-03
bta-miR-30f	-3.666	2.42E-47	3.53E-45
bta-miR-432	-3.720	2.43E-04	1.78E-03
bta-miR-370	-4.117	1.66E-04	1.32E-03
bta-miR-141	-4.856	2.21E-06	2.30E-05
bta-miR-200c	-5.178	1.95E-39	1.71E-37
bta-miR-133a	-5.754	2.64E-120	5.78E-118

S1 Table: List of the DE miRNAs in *Theileria*- infected leukocytes (TBL20)
