# Supplementary material for: miR-126-5p by direct targeting of JNK-interacting protein-2 (JIP-2) plays a key role in Theileria-infected macrophage virulence
Source: PLoS Pathog. 2018 Mar 23;14(3):e1006942. doi: 10.1371/journal.ppat.1006942 (PMC5892942; doi:10.1371/journal.ppat.1006942)
Supplement: S2 Table — (PDF) [file ppat.1006942.s003.pdf]

Column1	log2FoldChange	pvalue	padj
bta-miR-99a-5p	9.949	3.71E-91	5.75E-90
bta-miR-99a-3p	9.572	5.67E-08	1.98E-07
bta-miR-767	9.314	1.71E-07	5.65E-07
bta-miR-6526	9.282	1.78E-113	3.26E-112
bta-miR-100	9.093	1.36E-73	1.82E-72
bta-miR-125b	9.081	2.21E-272	1.37E-270
bta-miR-193b	8.452	4.85E-06	1.43E-05
bta-miR-346	8.347	7.03E-13	3.32E-12
bta-miR-449a	7.995	3.10E-24	2.23E-23
bta-miR-126-5p	7.902	2.73E-217	1.04E-215
bta-let-7c	7.204	2.06E-247	9.27E-246
bta-miR-449b	6.984	6.16E-09	2.28E-08
bta-miR-126-3p	6.941	3.55E-91	5.68E-90
bta-miR-105b	6.835	6.59E-04	1.66E-03
bta-miR-708	6.717	8.81E-04	2.20E-03
bta-miR-204	6.567	2.30E-26	1.73E-25
bta-miR-199b	6.380	1.54E-67	1.96E-66
bta-miR-105a	6.351	3.71E-03	8.52E-03
bta-miR-216b	6.068	1.95E-06	6.00E-06
bta-miR-218	5.875	3.98E-70	5.19E-69
bta-miR-449c	5.809	4.95E-06	1.45E-05
bta-miR-27a-3p	5.742	0.00E+00	0.00E+00
bta-miR-338	5.733	9.65E-29	7.48E-28
bta-miR-2461-3p	5.675	1.94E-24	1.42E-23
bta-miR-24-3p	5.465	1.50E-161	3.91E-160
bta-miR-146a	5.440	3.48E-211	1.23E-209
bta-miR-2904	5.252	3.20E-156	7.93E-155
bta-miR-7857	4.925	4.49E-30	3.65E-29
bta-miR-23a	4.601	5.95E-199	1.84E-197
bta-miR-2425-3p	4.265	1.52E-16	8.48E-16
bta-miR-2427	3.877	3.09E-13	1.52E-12
bta-miR-147	3.793	3.15E-37	2.79E-36
bta-miR-9-5p	-3.199	6.59E-13	3.14E-12
bta-miR-2316	-3.243	1.33E-10	5.90E-10
bta-miR-211	-3.498	1.10E-09	4.44E-09
bta-miR-210	-3.620	2.75E-136	5.93E-135
bta-miR-181b	-3.765	6.48E-255	3.57E-253
bta-miR-139	-3.882	2.68E-11	1.24E-10
bta-miR-122	-3.967	1.08E-04	2.89E-04
bta-miR-3660	-4.084	5.15E-24	3.65E-23
bta-miR-504	-4.236	6.94E-10	2.92E-09
bta-miR-2285t	-4.422	1.10E-110	1.94E-109
bta-miR-223	-4.695	3.20E-04	8.36E-04
bta-miR-486	-4.895	0.00E+00	0.00E+00
bta-miR-2300a-5p	-5.064	1.48E-02	3.15E-02
bta-miR-224	-5.095	1.41E-02	3.01E-02
bta-miR-181a	-5.181	0.00E+00	0.00E+00
bta-miR-95	-5.889	4.90E-37	4.26E-36
bta-miR-6121-3p	-5.899	2.79E-47	2.71E-46
bta-miR-551b	-6.038	2.36E-03	5.58E-03
bta-miR-599	-6.080	2.23E-03	5.28E-03
bta-miR-149-3p	-6.178	1.78E-03	4.30E-03
bta-miR-30a-5p	-6.322	0.00E+00	0.00E+00
bta-miR-149-5p	-6.712	0.00E+00	0.00E+00
bta-miR-2367-5p	-6.834	3.90E-04	1.01E-03
bta-miR-615	-6.902	3.22E-04	8.37E-04
bta-miR-150	-7.331	2.70E-32	2.27E-31
bta-miR-1	-7.466	7.08E-05	1.94E-04
bta-miR-135b	-7.636	4.34E-05	1.20E-04
bta-miR-30f	-7.873	0.00E+00	0.00E+00
bta-miR-133a	-8.041	1.55E-251	7.69E-250
bta-miR-196a	-8.481	2.34E-101	4.00E-100

S2 Table: List of the DE miRNAs in *Theileria*- infected leukocytes (TBL3)
